# *MdWRKY75e* enhances resistance to *Alternaria alternata* in *Malus domestica*

**DOI:** 10.1038/s41438-021-00701-0

**Published:** 2021-10-11

**Authors:** Yingjun Hou, Xinyi Yu, Weiping Chen, Weibing Zhuang, Sanhong Wang, Chao Sun, Lifang Cao, Tingting Zhou, Shenchun Qu

**Affiliations:** 1 College of Horticulture, Nanjing Agricultural University, Nanjing, People’s Republic of China; 2 College of Agriculture, Nanjing Agricultural University, Nanjing, People’s Republic of China; 3 Institute of Botany, Jiangsu Province and Chinese Academy of Sciences (Nanjing Botanical Garden, Memorial Sun Yat-sen), Nanjing, People’s Republic of China

**Keywords:** Molecular engineering, Molecular engineering in plants

## Abstract

The *Alternaria alternata* apple pathotype adversely affects apple (*Malus domestica* Borkh.) cultivation. However, the molecular mechanisms underlying enhanced resistance to this pathogen in apple remain poorly understood. We have previously reported that *MdWRKY75* expression is upregulated by *A. alternata* infection in ‘Sushuai’ apples. In this study, we discovered that overexpression of *MdWRKY75e* increased the resistance of transgenic apple lines to *A. alternata* infection, whereas silencing this gene enhanced susceptibility to *A. alternata* infection. Furthermore, we found that MdWRKY75e directly binds to the *MdLAC7* promoter to regulate the biosynthesis of laccase and increase the biosynthesis of lignin during *A. alternata* infection. Moreover, the thickening of the cell wall enhanced the mechanical defense capabilities of apple. In addition, we found that jasmonic acid remarkably induced *MdWRKY75e* expression, and its levels in transgenic apple lines were elevated. These results indicate that *MdWRKY75e* confers resistance to the *A. alternata* apple pathotype mainly via the jasmonic acid pathway and that pathogenesis-related genes and antioxidant-related enzyme activity are involved in the disease resistance of *MdWRKY75e* transgenic plants. In conclusion, our findings provide insights into the importance of *MdWRKY75e* for resistance to *A. alternata* infection in apples.

## Introduction

Apple (*Malus domestica*) is a globally cultivated fruit crop species. However, apple cultivation is adversely affected by various fungi, including those caused by the *Alternaria alternata* apple pathotype (*Alternaria alternata* f. sp. mali)^1–3^. Current *A. alternata* management practices include conventional chemical treatments with adverse side effects, such as environmental pollution and food safety hazards. The most effective strategy for fighting fungal diseases in apple is the selective breeding of resistant cultivars^2,4^. However, the mechanism of resistance to apple leaf spot disease in apples has not been extensively studied. Therefore, it is important to investigate the potential molecular mechanism underlying resistance to apple leaf spot disease.

Plants possess two major innate immune responses for defense against pathogens^5^. One response involves pattern recognition receptors (PRRs) that recognize pathogen-associated molecular patterns (PAMPs), and the stimulation of PRRs results in PAMP-triggered immunity to activate a basal defense response; the other involves effector-triggered immunity, which induces the expression of resistance gene products that specifically recognize the effectors delivered by pathogens to weaken PAMP-triggered immunity^6–9^. The two innate immune systems partially work together to trigger downstream responses, such as the generation of plant hormones, reactive oxygen species (ROS), and secondary metabolites, and the induction of a hypersensitive response to activate the corresponding defense systems^10^.

Various plant hormones, such as salicylic acid (SA), ethylene (ET), and jasmonic acid (JA), enhance plant defense responses against pathogens and abiotic stress^11,12^. SA is principally involved in systemic acquired resistance as a mediator of plant defense responses to biotrophic and hemibiotrophic pathogens, while JA and ET interact with each other to activate defense responses against necrotrophic pathogens. The interactions between JA and SA signaling pathways are antagonistic rather than cooperative^13^. Both the SA and JA pathways are stimulated by pathogen infection, leading to the accumulation of endogenous SA and JA, which act as signaling molecules for transcriptional responses of pathogen-associated genes^14,15^. JASMONATE INSENSITIVE 1/MYC2 (JIN1/MYC2), a transcription factor, mediates JA-responsive gene expression to trigger defense responses in host plants^16^. The JA-responsive marker genes thionin (*Thi2.1*) and defensin (*PDF1.2*) accumulate in plants challenged by fungal pathogens^17,18^. Under biotic and abiotic stresses, plants can improve the activities of antioxidant enzymes, such as catalase (CAT), superoxide dismutase (SOD), and peroxidase (POD), which scavenge ROS, particularly H_2_O_2_ and O_2_^−^, to reduce their accumulation and mitigate their damage by maintaining dynamic equilibrium^19,20^. The enzyme phenylalanine ammonia-lyase (PAL) is critical for SA synthesis pathways that limit biotic and abiotic stresses^21^. Additionally, PAL has an important role in lignin synthesis via the phenylpropanoid pathway, which is the most common secondary metabolic pathway involved in plant defense reactions against abiotic and biotic stresses; the polymerization of monolignol to form lignin provides mechanical strength and reinforces plant cell walls, providing a physical barrier against pathogens and pests^22,23^. The polymerization of monolignols into lignin polymers is catalyzed either by peroxidases or laccases^24^. In apple, the molecular mechanisms underlying the genetic responses conferring resistance to fungal infections are still unclear.

The WRKY transcription factor family in plants plays an important role in signaling pathways that respond to biotic (fungi, pathogenic bacteria, oomycetes, and viruses) and abiotic (heat, soil salinity, oxidative stress, drought, cold, irradiation, and heavy metals) stresses. The WRKY protein structural domain comprises a 60-amino acid-long DNA bonding domain characterized by a highly conserved N-terminal core known as the WRKYGQK motif. WRKYs are divided into three major groups (I, II, and III), where group II is further categorized into five subgroups (IIa, IIb, IIc, IId, and IIe) according to the number of WRKY structural domains and the structure of the zinc finger motif^25,26^. Studies on *Arabidopsis* have revealed that *AtWRKY75* overexpression enhances resistance to *Pectobacterium carotovora* ssp. *Carotovora* (Pcc) via SA and JA defense signaling^27^. Similarly, *AtWRKY75* decreases the severity of Pcc-induced bacterial soft rot and activates a subset of defense-related genes against Pcc infection in Chinese cabbage^28^. *AtWRKY75* and *AtWRKY28* overexpression strengthens the resistance to oxalic acid and S*clerotinia sclerotiorum* stress, mainly via the JA/Eth pathway^29^. AtWRKY75 interacts with DELLA proteins, which affect flowering via the GA-mediated signaling pathway in *Arabidopsis*^30^ and induce leaf senescence by interacting with SA and ROS^31^. *PtrWRKY75* overexpression enhances drought tolerance through salicylic acid-induced ROS accumulation in poplar^32^. Additionally, *PagWRKY75* can negatively regulate salt and osmotic tolerance by modulating various physiological processes in poplar^33^. In apple, *MdWRKY100* overexpression increases resistance to *Colletotrichum gloeosporioides*, while its silencing by RNAi rendered the transgenic plants more sensitive to this pathogen^34^. *MdWRKY33* contributes to resistance against *Pythium ultimum* infection in apple roots^35^. MdWRKY15, MdWRKY31, and MdWRKY46 enhance resistance to *Botryosphaeria dothidea* in apple by activating the expression of *MdICS1*, *MdHIR4*, and *MdPBS3.1*, respectively, in the SA biosynthesis pathway^36–38^.

Herein, we found that *MdWRKY75e* was significantly induced by *A. alternata* and MeJA treatments. However, *MdWRKY75e* expression was significantly inhibited by SA treatment, and *MdWRKY75e* was directly bound to the *MdLAC7* gene promoter. We further show that MdWRKY75e is a positive regulator of *MdLAC7* expression as well as laccase and lignin biosynthesis. Moreover, our findings suggest that MdWRKY75e acts as a positive modulator of disease resistance, at least in part, by regulating antioxidant enzyme-mediated ROS scavenging. Our study reveals the molecular mechanism underlying the role of WRKYs in apple resistance to fungal diseases and provides a candidate gene for breeding disease-resistant apples.

## Results

### Isolation and bioinformatics analysis of *MdWRKY75s*

**Fig. 1:**
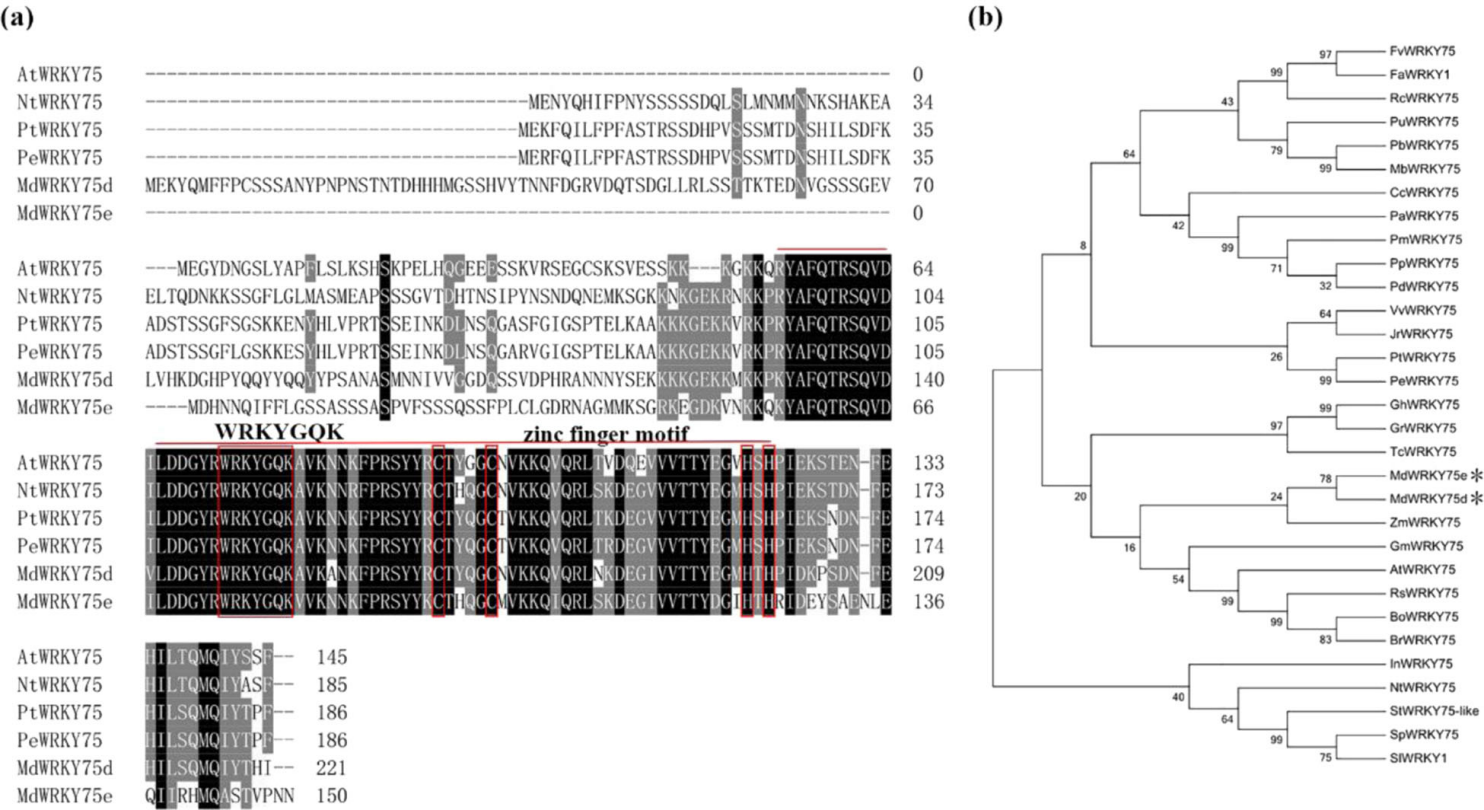
Multiple alignments of MdWRKY75d and MdWRKY75e and phylogenetic analysis of WRKY75s from different plants. **a** Amino acid sequence alignment of MdWRKY75d, MdWRKY75e, and WRKY75s from various plant species. The same amino acids are displayed on a black background, and similar amino acids are shown in gray. The WRKY motif is indicated by a line, and the zinc finger structure is marked by a red circle. The sequences used for comparison with MdWRKY75d and MdWRKY75e were *Arabidopsis thaliana* AtWRKY75 (NP_196812.1), *Nicotiana tabacum* NtWRKY75 (XP_016446514.1), *Populus trichocarpa* PtWRKY75 (XP_006373216.1), and *Populus euphratica* PeWRKY75 (XP_011009191.1). **b** Construction of a phylogenetic tree on the basis of WRKYs from different plants, including MdWRKY75d and MdWRKY75e. A phylogenetic tree was created with MdWRKY75d, MdWRKY75e, and the following WRKY75 family proteins: *Vitis vinifera* VvWRKY75 (XP_002275576.1), *Populus trichocarpa* PtWRKY75 (XP_006373216.1), *Populus euphratica* PeWRKY75 (XP_011009191.1), *Nicotiana tabacum NtWRKY75* (XP_016446514.1), *Arabidopsis thaliana* AtWRKY75 (NP_196812.1), *Raphanus sativus RsWRKY75* (XP_018475395.1), *Brassica oleracea* var. oleracea BoWRKY75 (XP_013626795.1), *Brassica rapa* BrWRKY75 (XP_009131315.1), *Rosa chinensis RcWRKY75* (XP_024197588.1), *Fragaria vesca* subsp. vesca FvWRKY75 (XP_004310100.1), *Pyrus × bretschneideri* PbWRKY75 (XP_009372253.1), *Fragaria × ananassa* FaWRKY1 (ACH88751.1), *Ipomoea nil* InWRKY75 (XP_019199847.1), *Theobroma cacao* TcWRKY75 (XP_007031915.1), *Malus baccata* MbWRKY75 (AHJ78583.1), *Juglans regia* JrWRKY75 (XP_018836802.1), *Prunus avium* PaWRKY75 (XP_021828982.1), *Gossypium hirsutum* GhWRKY75 (XP_016725404.1), *Pyrus ussuriensis × Pyrus communis* PuWRKY75 (KAB2599104.1), *Gossypium raimondii* GrWRKY75 (XP_012446088.1), *Prunus mume* PmWRKY75 (XP_008222197.1), *Prunus persica* PpWRKY75 (XP_007226197.1), *Prunus dulcis PdWRKY75* (XP_034198908.1), *Citrus clementina* CcWRKY75 (XP_006437986.1), *Solanum tuberosum* StWRKY75-like (NP_001275604.1), *Solanum pennellii* SpWRKY75 (XP_015075730.1), *Solanum lycopersicum* SlWRKY1 (NP_001310244.1), *Glycine max* GmWRKY75 (XP_003549123.1), and *Zea mays* ZmWRKY75 (PWZ27788.1). Bootstrap values are shown for each diverging branch. MdWRKY75d and MdWRKY75d are denoted with asterisks

We previously identified the infection-induced WRKY TFs *MdWRKY75d* and *MdWRKY75e* in the transcriptome of ‘Sushuai’ apple. *MdWRKY75d* sequencing revealed that the full length of the cDNA was 666 bp. Bioinformatics analysis indicated that *MdWRKY75d* encodes a putative peptide consisting of 221 amino acids, with an isoelectric point of 9.12 and a molecular mass of 25.5 kDa. *MdWRKY75e* sequencing showed that the full length of its cDNA was 453 bp, and bioinformatics analysis suggested that it encoded a putative peptide composed of 150 amino acids, with an isoelectric point of 9.56 and a molecular mass of 17.1 kDa. Multiple sequence alignment suggested that MdWRKY75d and MdWRKY75e had a highly conserved sequence, including a WRKYGQK structural domain followed by a zinc finger-like motif (Fig. 1a). The phylogenetic tree constructed with MdWRKY75d and MdWRKY75e, as well as a total of 29 WRKYs from different plants, showed that these TFs are most closely related to ZmWRKY75 (Fig. 1b).

### Expression of *MdWRKY75s* under different treatments and in various organisms

The expression patterns of *MdWRKY75d* and *MdWRKY75e* under infection with pathogens, MeJA and SA treatments were examined by RT–qPCR (unpublished article). As shown in Supplementary Fig. S1a–c, *MdWRKY75d* and *MdWRKY75e* transcript levels were significantly induced by *A. alternata* and MeJA treatment. However, *MdWRKY75d* and *MdWRKY75e* expression was significantly inhibited by SA treatment.

*MdWRKY75d* and *MdWRKY75e* transcripts were detected in all organs of the apple plants. The abundance of *MdWRKY75d* transcripts was high in the flowers and ripe fruits, moderate in the stems and young fruits, and low in young and old leaves. Conversely, the abundance of *MdWRKY75e* transcripts was high in young leaves, moderate in old leaves, ripe fruits, and flowers, and low in stems and young fruits (Supplementary Fig. S1d).

### Subcellular localization of MdWRKY75s

**Fig. 2:**
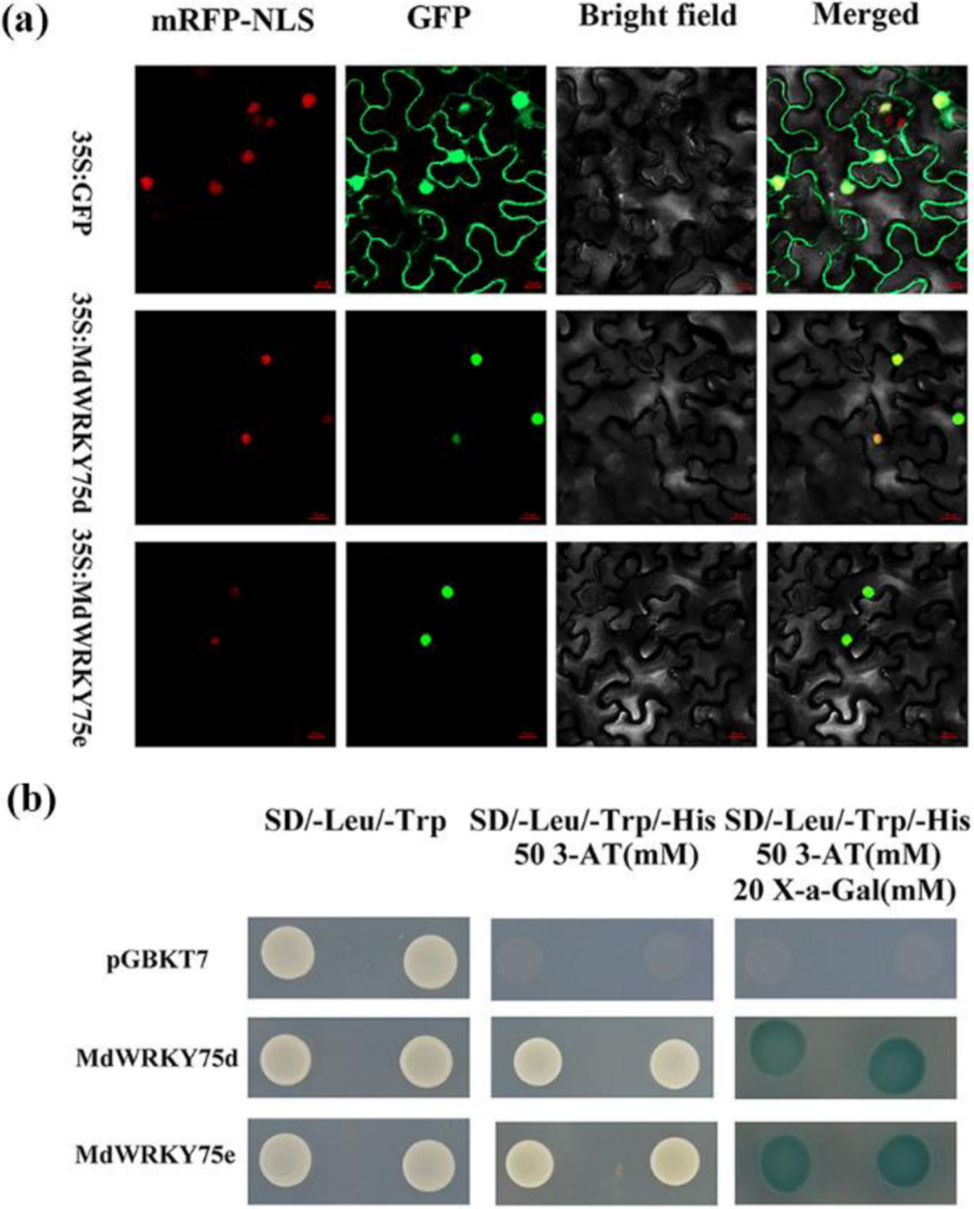
Subcellular localization of MdWRKY75d and MdWRKY75e in *N. benthamiana* and transcriptional activity of MdWRKY75d and MdWRKY75e in a yeast assay system. **a** Tobacco leaves were transformed with constructs including control (GFP) or fusion plasmids (*MdWRKY75::GFP*). The images correspond to mRFP-NLS (left), GFP, bright field (middle), and merged (right). 35S::D53-RFP was utilized as a nuclear marker (mRFP-NLS). Bars: *35S::GFP*, 50 μm; *35S::MdWRKY75d* and *35S::MdWRKY75d*, 20 μm. **b** Yeast cells (strain AH109) transformed with the control vector (top panel) or the fusion vector-containing MdWRKY75 (middle and bottom panels) were grown on SD/-Leu/-Trp or SD/-Leu/-Trp/-His with or without 3-AT and 20 mM X-a-gal

Fusion plasmids for *35S::GFP-MdWRKY75d* and *35S::GFP-MdWRKY75e* were generated by fusing the full length of *MdWRKY75d* and *MdWRKY75e* to the N terminus of the GFP reporter protein using the pCAMBIA1302 vector driven by the CaMV 35S promoter. Next, *35S::GFP-MdWRKY75d*, *35S::GFP-MdWRKY75e*, and a nuclear marker (35S::D53-RFP construct; positive control) were cotransformed into *Nicotiana benthamiana* leaves by *Agrobacterium-*mediated transient transformation^39^. Microscopic observation showed that the *35S::GFP* vector was present in the cytoplasm and nucleus of tobacco cells, while the *MdWRKY75d-GFP* and *MdWRKY75e-GFP* fusion proteins were only present in the nucleus (Fig. 2a). Together, these data suggest that MdWRKY75d and MdWRKY75e localize to the nucleus.

### Transactivation activity of MdWRKY75s

The fusion plasmids were generated by fusing the full-length coding regions of *MdWRKY75d* and *MdWRKY75e* with the DNA binding domain of GAL4 using the pGBKT7 vector and transformed into the yeast strain AH109. The growth of the fusion plasmid-containing yeast cells was comparable to that of yeast containing the control plasmid (pGBKT7) in the same selection medium (SD)/Leu/Trp. Yeast cells transformed with any vector grew on SD/-Leu/-Trp medium, showing that this system was reliable. Yeast cells transformed with the negative control plasmid pGBKT7 failed to grow on SD/-Leu/-Trp/-His medium, while cells transformed with the reconstituted vector survived on a selection medium supplemented with 50 mM 3-amino-1,2,4-triazole (3-AT). Furthermore, only yeast cells transformed with the fusion plasmid changed to blue when cultivated on SD/Leu/Trp/His medium supplemented with both 50 mM 3-AT and 20 mM X-a-Gal (Fig. 2b). These results indicated that MdWRKY75d and MdWRKY75e have transactivation activity in yeast.

### Overexpression of *MdWRKY75e* modulated laccase biosynthesis and altered the endogenous lignin contents in transgenic plants

Under the control of the CaMV 35S promoter, *MdWRKY75d-* and *MdWRKY75e*-overexpressing transgenic tobacco was generated by *Agrobacterium*-mediated transformation (Supplementary Fig. S2A). In total, eight transformants (T_0_ generation) of *MdWRKY75d* and *MdWRKY75e* were confirmed as positive lines by GUS staining. Semiquantitative RT–PCR analysis showed that *MdWRKY75d* and *MdWRKY75e* were overexpressed in six and seven lines, respectively. Based on the real-time qPCR results (Supplementary Table S1), three independent overexpression lines of tobacco from among the six *MdWRKY75d*-overexpressing lines, designated #18, #25, and #36, and another three from the seven *MdWRKY75e*-overexpressing lines, named #9, #32, and #36, were selected for the following assays, together with the corresponding untransformed wild-type (WT) and empty vector-transformed WT (empty vector) lines (Supplementary Fig. S2B, C).

**Fig. 3:**
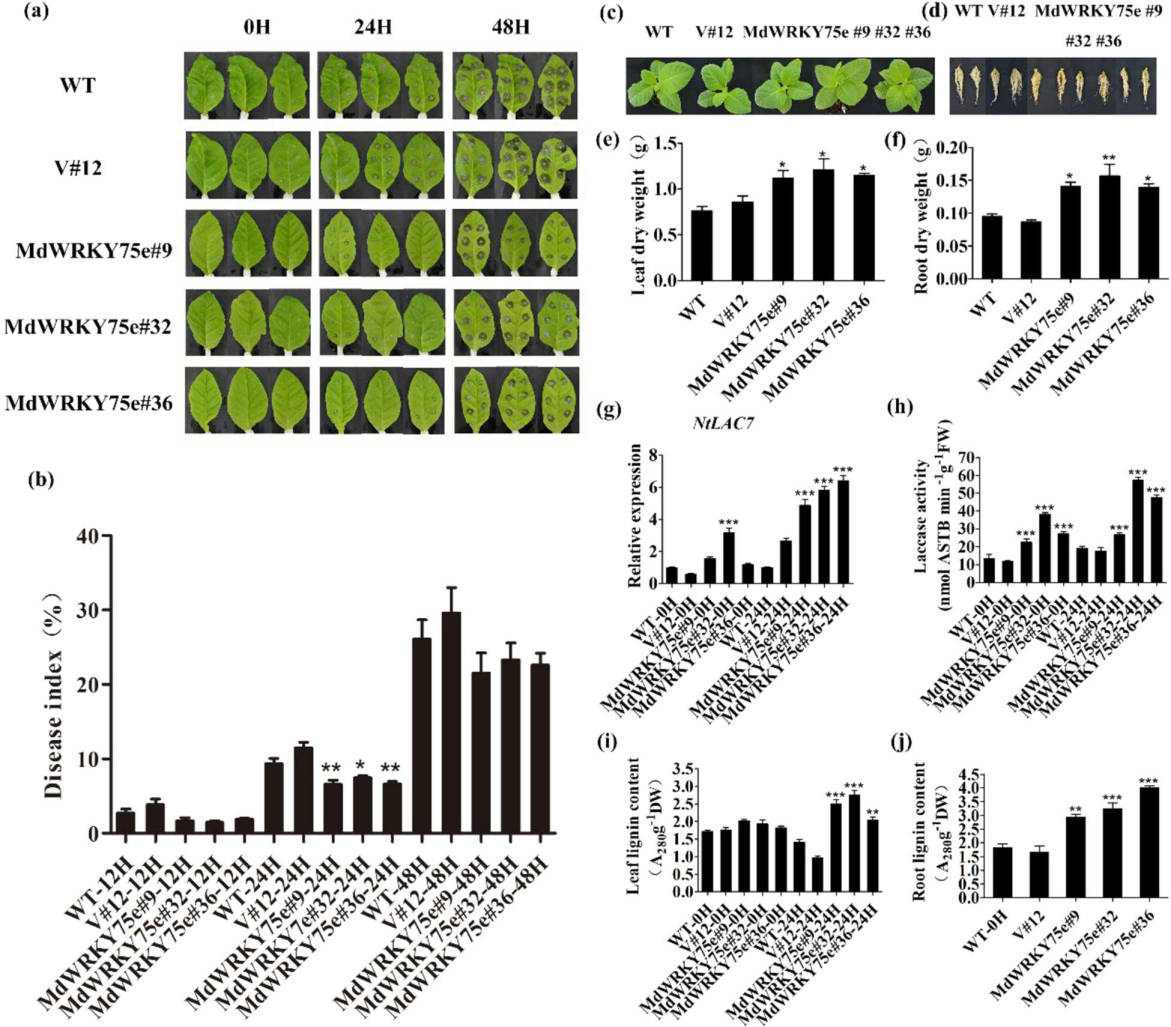
Overexpression of *MdWRKY75e* conferred enhanced resistance to *B. cinerea* infection in transgenic tobacco. **a** Time course of *B. cinerea* infection of WT, V#12, and *MdWRKY75e* transgenic lines (#9, #32, and #36) for 48 h for phenotype analysis. **b** Time course of *B. cinerea* infection of WT, V#12, and *MdWRKY75e* transgenic lines for 48 h for disease index analysis. **c**, **d** Phenotypes of plants (**c**) and root (**d**) systems in tobacco WT, V#12, and *MdWRKY75e* transgenic lines. **e**, **f** Results of leaf (**e**) and root (**f**) dry weight measurements in tobacco WT, V#12, and *MdWRKY75e* transgenic lines. **g**–**i** Expression of *NtLAC7* (**g**), activity of laccase (**h**), and lignin content of the leaves (**i**) in tobacco WT, V#12, and *MdWRKY75e* transgenic lines after 0 and 24 h of *B. cinerea* infection. **j** Root lignin content in tobacco WT, V#12, and *MdWRKY75e* transgenic lines. WT: wild-type; V#12: empty vector; and *MdWRKY75e*#9, *MdWRKY75e*#32, and *MdWRKY75e*#36: *MdWRKY75e* transgenic lines

Forty-five-days-old WT, empty vector, and transgenic plants with consistent growth were infected with the pathogen for 48 h. Interestingly, the WT and empty vector plants showed greater sensitivity to infection with pathogens than *MdWRKY75e* transgenic plants. In particular, lesion sizes in *MdWRKY75e* transgenic lines were particularly small after 24 h of infection with pathogens, and this result was further confirmed by the disease index (Fig. 3a, b). In contrast, lesion sizes in *MdWRKY75d* transgenic plants were not significantly different compared to those in the WT and empty vector plants (Supplementary Fig. S3a, b). Therefore, we further found that the growth phenotypes and root lengths of *MdWRKY75e* transgenic plants were not markedly different from those of the WT and empty vector plants (Fig. 3c). Compared to the WT and empty vector plants, the roots of transgenic *MdWRKY75e* plants had a deeper color and increased lignification (Fig. 3d). Furthermore, the dry weights of leaves and roots were heavier in transgenic *MdWRKY75e* plants than in the WT and empty vector plants (Fig. 3e, f). As shown in Fig. 3g–j, *NtLAC7* expression and laccase activity in *MdWRKY75e* transgenic plants were significantly higher than those in WT and empty vector plants. Moreover, the lignin contents in the leaves and roots of *MdWRKY75e* transgenic lines were markedly higher than those of WT and empty vector plants.

**Fig. 4:**
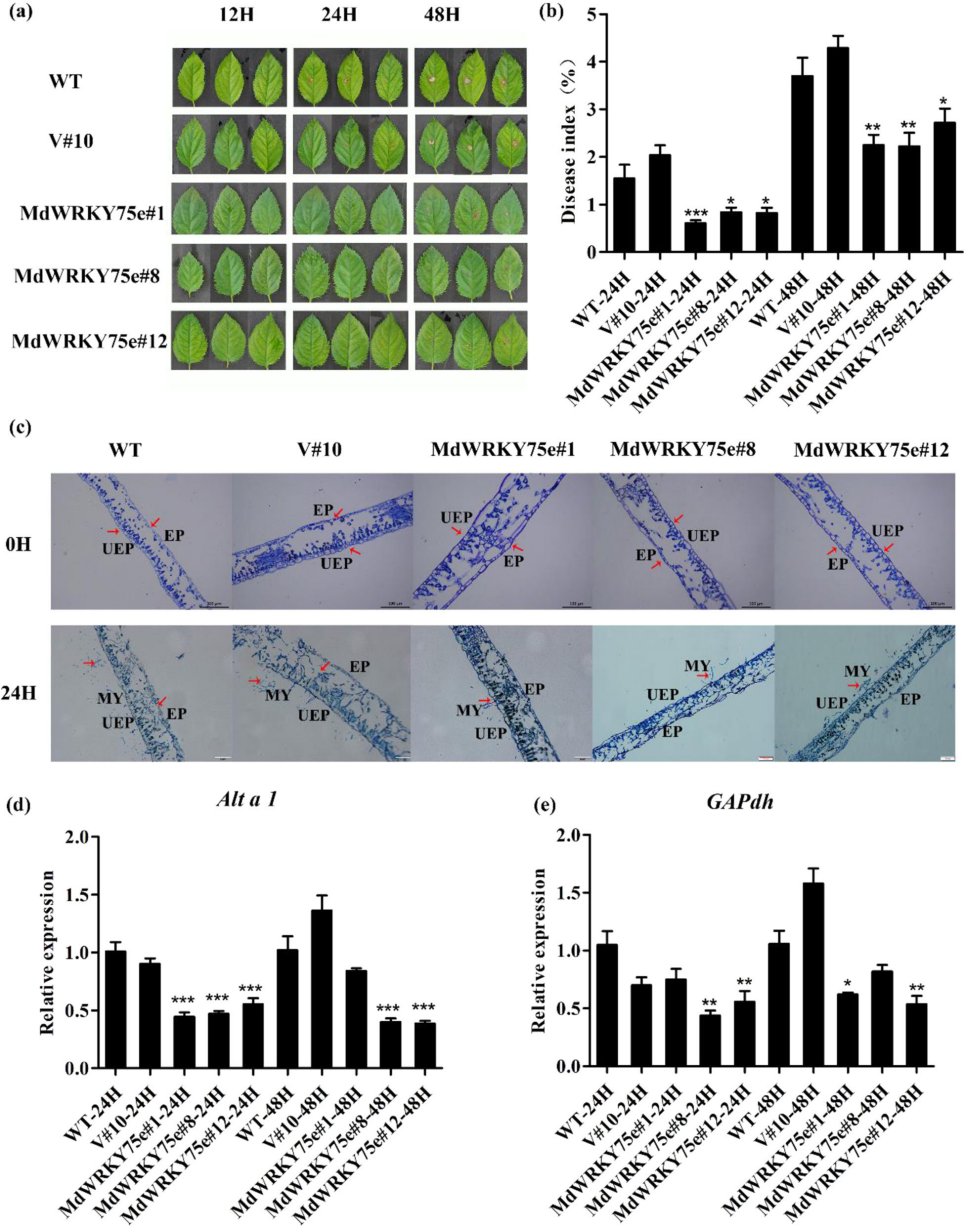
Overexpression of *MdWRKY75e* enhanced *A. alternata* infection in transgenic apple. **a** Time course of *A. alternata* infection of WT, V#10, and *MdWRKY75e* transgenic lines (#1, #8, and #12) for 48 h for phenotype analysis. **b** Time course of *A. alternata* infection of WT, V#10, and *MdWRKY75e* transgenic lines for 48 h for disease index analysis. **c** Phenotypes of paraffin sections of leaves infected with *A. alternata* at 0 and 24 h in WT, V#10, and *MdWRKY75e* transgenic lines. UEP upper epidermis, EP epidermis, and MY mycelium. **d**, **e** Time course of the expression of *Alta1* (**d**) and *GAPdh* (**e**) genes in WT, V#10, and *MdWRKY75e* transgenic lines under *A. alternata* infection for 48 h. *GAPdh* and *Alta1* in the internal transcribed spacer region sequences of *Alternaria species* can be used as marker genes for the severity of *A. alternata* apple pathotype infection^40^. WT: wild-type; V#10: empty vector; and *MdWRKY75e*#1, *MdWRKY75e*#8, and *MdWRKY75e*#12: *MdWRKY75e* transgenic lines

Three transgenic GL-3 lines (designated #1, #8, and #12) (Supplementary Fig. S4A) were examined by GUS staining, semi-quantitative RT–PCR, and qRT–PCR analyses (Supplementary Fig. S4B). Exposure of 45-days-old WT, empty vector, and transgenic GL-3 plants (Supplementary Fig. S4C) to disease stress caused by infection with pathogens for 48 h showed that the WT and empty vector plants had significantly larger lesion areas in leaves than the transgenic plants, further confirming the disease index results (Fig. 4a, b). Paraffin sectioning with toluidine blue O staining to compare the physiological differences among transgenic, WT, and empty vector plants showed that the upper epidermal cell wall was thickened in the transgenic line but not in the others. Moreover, wound and mycelium formation in transgenic lines was significantly smaller than that in the WT and empty vector plants after 24 h of infection with pathogens (Fig. 4c). Moreover, the expression levels of *Alta1* and *GAPdh* in *A. alternata* were considerably lower in the transgenic plants than in the WT and empty vector plants (Fig. 4d, e).

**Fig. 5:**
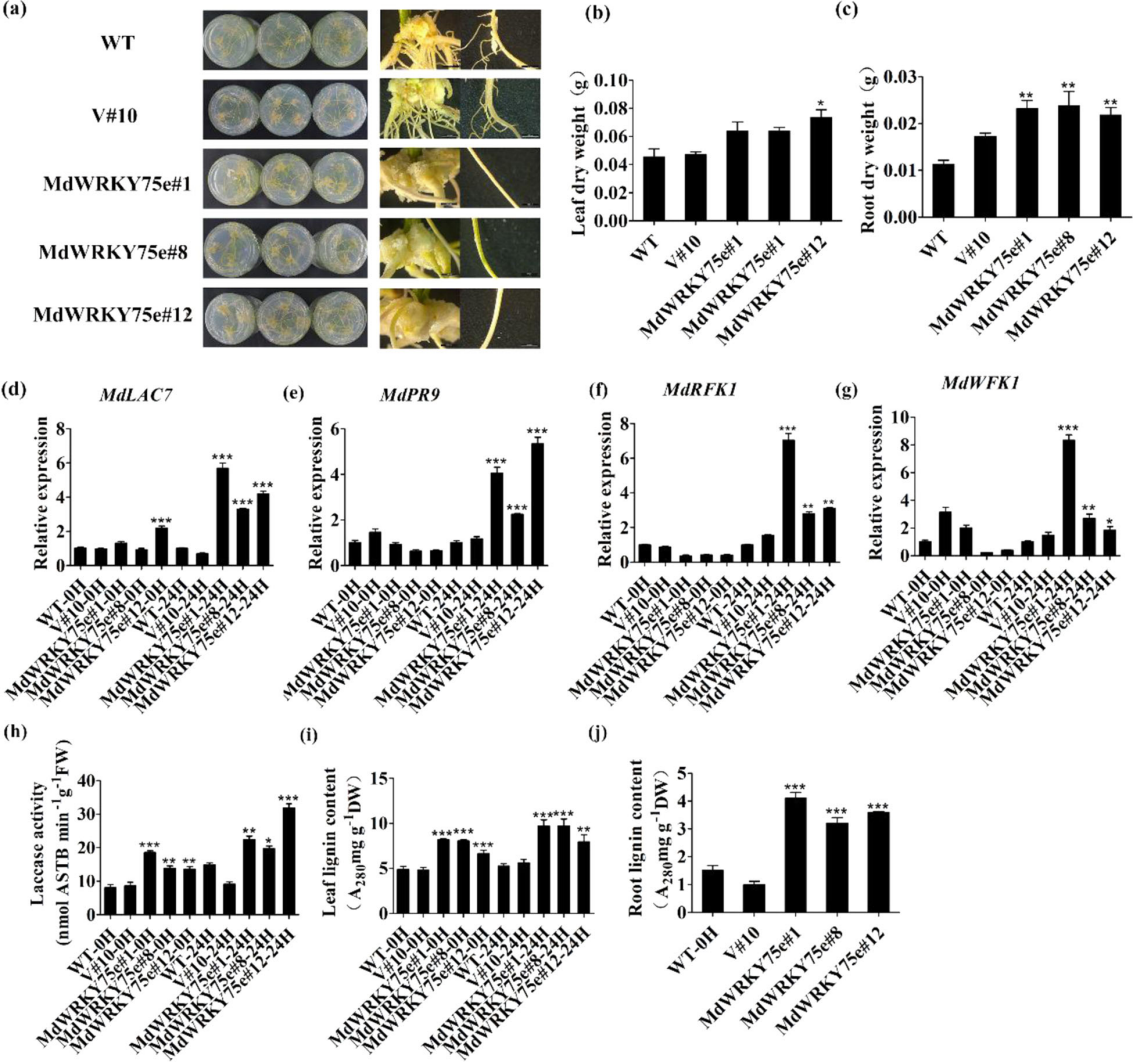
Phenotypic analysis of *MdWRKY75e* transgenic apple plants. **a** Phenotypes of roots in WT, V#10, and *MdWRKY75e* transgenic lines (#1, #8, and #12). **b**, **c** Results of leaf (**b**) and root (**c**) dry weight measurements in WT, V#10, and *MdWRKY75e* transgenic lines. **d**–**g** Expression of the MdWRKY target genes *MdLAC7* (**d**), *MdWRK1* (**e**), *MdFRK1* (**f**), and *MdPR9* (**g**) in WT, V#10, and *MdWRKY75e* transgenic lines at 0 and 24 h of infection. **h**, **i** Laccase activity (**h**) and leaf lignin content (**i**) in WT, V#10, and *MdWRKY75e* transgenic lines at 0 and 24 h of infection with *A. alternata*. **j** Root lignin content in WT, V#10, and *MdWRKY75e* transgenic lines. WT: wild-type; V#10: empty vector; and *MdWRKY75e*#1, *MdWRKY75e*#8, and *MdWRKY75e*#12: *MdWRKY75e* transgenic lines

Interestingly, root callus formation and lignification in transgenic plants were noticeably higher than those in WT and empty vector plants, consistent with the growth phenotype. Furthermore, *MdWRKY75e* overexpression suppressed root hair development, lateral root length and number, and root hair number (Fig. 5a). We further found that root dry weight was higher in transgenic plants than in WT and empty vector plants; in contrast, leaf dry weight was slightly higher in transgenic plants than in WT and empty vector plants (Fig. 5b, c). *MdLAC7*, *MdPR9*, *MdRFK1*, and *MdWRK1* expression was strongly induced in *MdWRKY75e*-overexpressing lines after pathogen infection (Fig. 5d–g). Furthermore, we attempted to determine whether laccase activities were altered in the tested plants and found them to be progressively higher in the transgenic lines than in the WT and empty vector lines (Fig. 5h). Additionally, we found that the lignin contents of leaves and roots in GL-3 transgenic lines were remarkably higher than those in WT and empty vector plants (Fig. 5i, j). In summary, these data showed that *MdWRKY75e* overexpression improved endogenous laccase activities and lignin contents and enhanced disease tolerance in transgenic plants.

### *MdWRKY75e* silencing in GL-3 confers sensitivity to pathogen infection

**Fig. 6:**
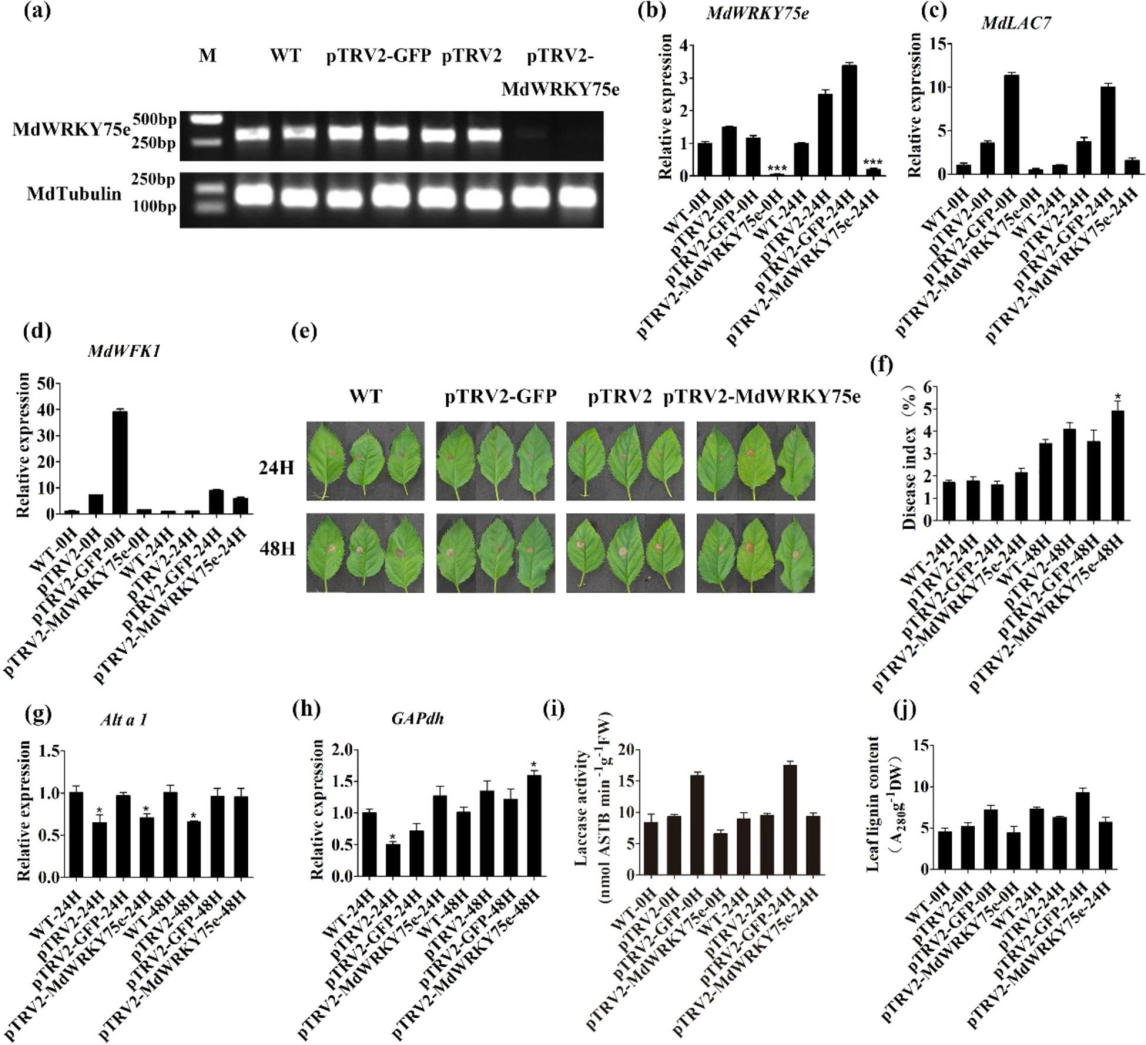
Silencing of *MdWRKY75e* by virus-induced gene silencing (VIGS) resulted in improved infection sensitivity in apple. **a** Semiquantitative RT–PCR detection of *MdWRKY75e* gene silencing efficiency. **b**–**d** Time course of the expression of the *MdWRKY75e* (**b**), *MdLAC7* (**c**), and *MdWFRK1* (**d**) genes in WT, pTRV2-*GFP*, pTRV2, and pTRV2-*MdWRKY75e* at 24 h. **e** Time course of *A. alternata* infection of WT, pTRV2-*GFP*, pTRV2, and pTRV2-*MdWRKY75e* for 48 h for phenotype analysis. **f** Time course of *A. alternata* infection of WT, pTRV2-*GFP*, pTRV2, and pTRV2-*MdWRKY75e* for 48 h for disease index analysis. **g**, **h** Time course of the expression of *Alta1* (**g**) and *GAPdh* (**h**) in WT, pTRV2-*GFP*, pTRV2, and pTRV2-*MdWRKY75e* after *A. alternata* infection for 48 h. **i**, **j** Laccase activity (**i**) and leaf lignin content (**j**) in WT, pTRV2-*GFP*, pTRV2, and pTRV2-*MdWRKY75e* at 0 and 24 h after *A. alternata* infection

We attempted to knock down *MdWRKY75e* in GL-3 transgenic lines by virus-induced gene silencing (VIGS). We constructed a pTRV2-*GFP* vector and reliably transfected apples with the pTRV2 system (Supplementary Fig. S5a). Semiquantitative RT–PCR was used to identify the efficiency of gene silencing and showed that the expression of *MdWRKY75e* was extremely low (Fig. 6a). Transcript analysis of young leaves further revealed that the transcription of *MdWRKY75e*, *MdLAC7*, and *MdWFK1* was inhibited in the silenced plants (Fig. 6b–d); however, the expression of *MdPR9* and *MdRFK1* did not change notably in the silenced plants (Supplementary Fig. S5b, c). We selected six MdWRKY genes that have close evolutionary relationships with MdWRKY75e to verify whether these gene expression levels were affected in *MdWRKY75e* knockdown lines. Our results showed that the expression levels of these genes were not significantly different in *MdWRKY75e* knockdown lines (Supplementary Fig. S5d–i). The above results indicated that the *MdWRKY75e* gene was silenced and did not affect the expression of other *MdWRKYs*. We next compared the levels of disease resistance in leaves of in vitro silenced plants 48 h after infection with pathogens and found slight differences in pTRV2-*MdWRKY75e* leaves compared with WT, empty vector (pTRV2), and pTRV2-*GFP* leaves (Fig. 6e). Time-course analyses of the disease index and *GAPdh* expression demonstrated greater disease susceptibility in pTRV2-*MdWRKY75e* leaves than in WT, empty vector (pTRV2), and pTRV2-*GFP* leaves. Time-course analysis of *Alta1* expression did not reveal remarkable changes in pTRV2-*MdWRKY75e* leaves when compared with WT, empty vector (pTRV2), and pTRV2-*GFP* leaves (Fig. 6f–h). Similarly, laccase activity and lignin content were not significantly different in pTRV2-*MdWRKY75e* leaves, although these levels were somewhat lower than those in WT, empty vector (pTRV2), and pTRV2-*GFP* leaves (Fig. 6i, j). These findings suggested that silencing *MdWRKY75e* by VIGS increased *A. alternata* susceptibility in GL-3 transgenic plants.

### Phenotype and endogenous hormone contents in transgenic apple lines overexpressing *MdWRKY75e*

The three GL-3 transgenic lines (#1, #8, and #12) exhibited dwarfism, uniform serration of the leaf edges, a reduced leaf shape index, and a greener leaf color after 2 months of transplantation (Supplementary Figs. S4C and S6A, B).

SA and JA levels were quantitatively measured in transgenic plants. As shown in Supplementary Fig. S7A, B, JA and methyl jasmonate levels were slightly higher, whereas SA and methyl salicylate levels were remarkably lower in the transgenic lines than in the WT and empty vector lines. Moreover, administration of exogenous 0.1 mM MeJA and SA to WT, empty vector, and transgenic lines revealed that MeJA treatment significantly increased the expression of *MdMYC2* in transgenic lines compared to that in WT and empty vector lines, although the level of *MdNPR3* transcripts in transgenic lines did not change. SA treatment marginally increased the level of *MdNPR3* transcripts in transgenic lines compared to that in WT and empty vector lines but did not affect *MdMYC2* expression.

### Transgenic lines accumulate lower ROS and display higher antioxidant enzyme activities after infection with pathogens

**Fig. 7:**
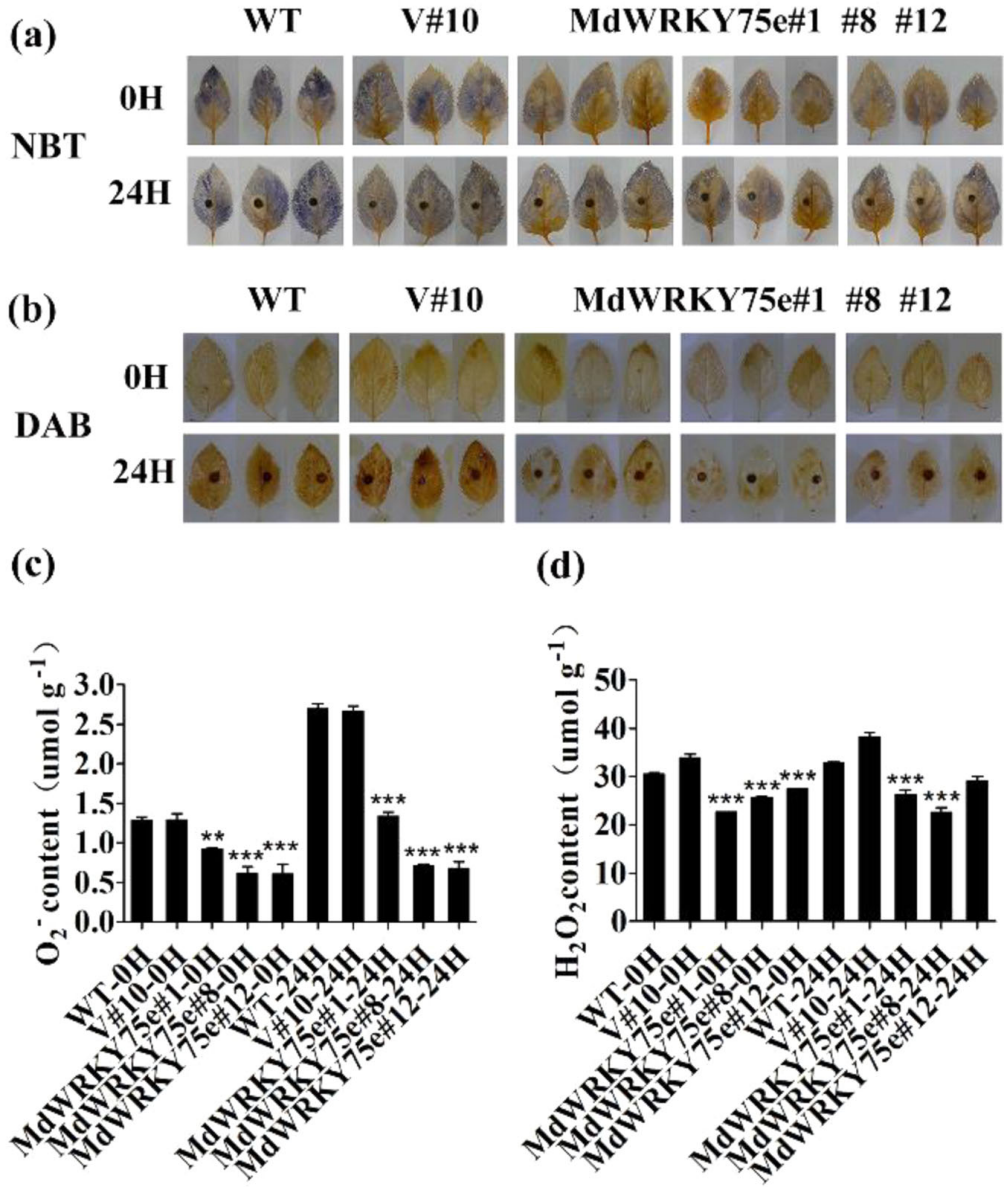
Analysis of O_2_^-^ and H_2_O_2_ in transgenic apple plants at 0 and 24 h after *A. alternata* infection. **a**, **b** Histochemical staining with DAB and NBT for O_2_^−^ (**a**) and H_2_O_2_ (**b**) in situ accumulation, respectively, in WT, V#10, and *MdWRKY75e* transgenic lines (#1, #8, and #12) at 0 and 24 h of infection. **c**, **d** Levels of O_2_^−^ (**c**) and H_2_O_2_ (**d**) in WT, V#10, and *MdWRKY75e* transgenic lines at 0 and 24 h of infection. WT: wild-type; V#12: empty vector; and *MdWRKY75e*#1, *MdWRKY75e*#8, and *MdWRKY75e*#12: *MdWRKY75e* transgenic lines

Histochemical staining with nitro blue tetrazolium (NBT) and diaminobenzidine (DAB) showed the levels of O_2_^−^ and H_2_O_2_, respectively, in the leaves of plants 24 h after infection with pathogens. As shown in Fig. 7a, b, the NBT staining intensity in WT and empty vector leaves was stronger than that in transgenic leaves. DAB staining revealed evenly distributed brown precipitates all over the leaves of WT and empty vector plants, whereas the leaves of the transgenic plants were only lightly stained, indicating lower O_2_^−^ and H_2_O_2_ generation after infection with pathogens. Quantitative measurements further showed that the H_2_O_2_ and O_2_^−^ contents in the GL-3 transgenic lines were particularly lower than those in the WT and empty vector lines (Fig. 7c, d). Thus, histochemical staining and quantitative determinations demonstrated that the transgenic plants were more resistant to oxidative stress.

**Fig. 8:**
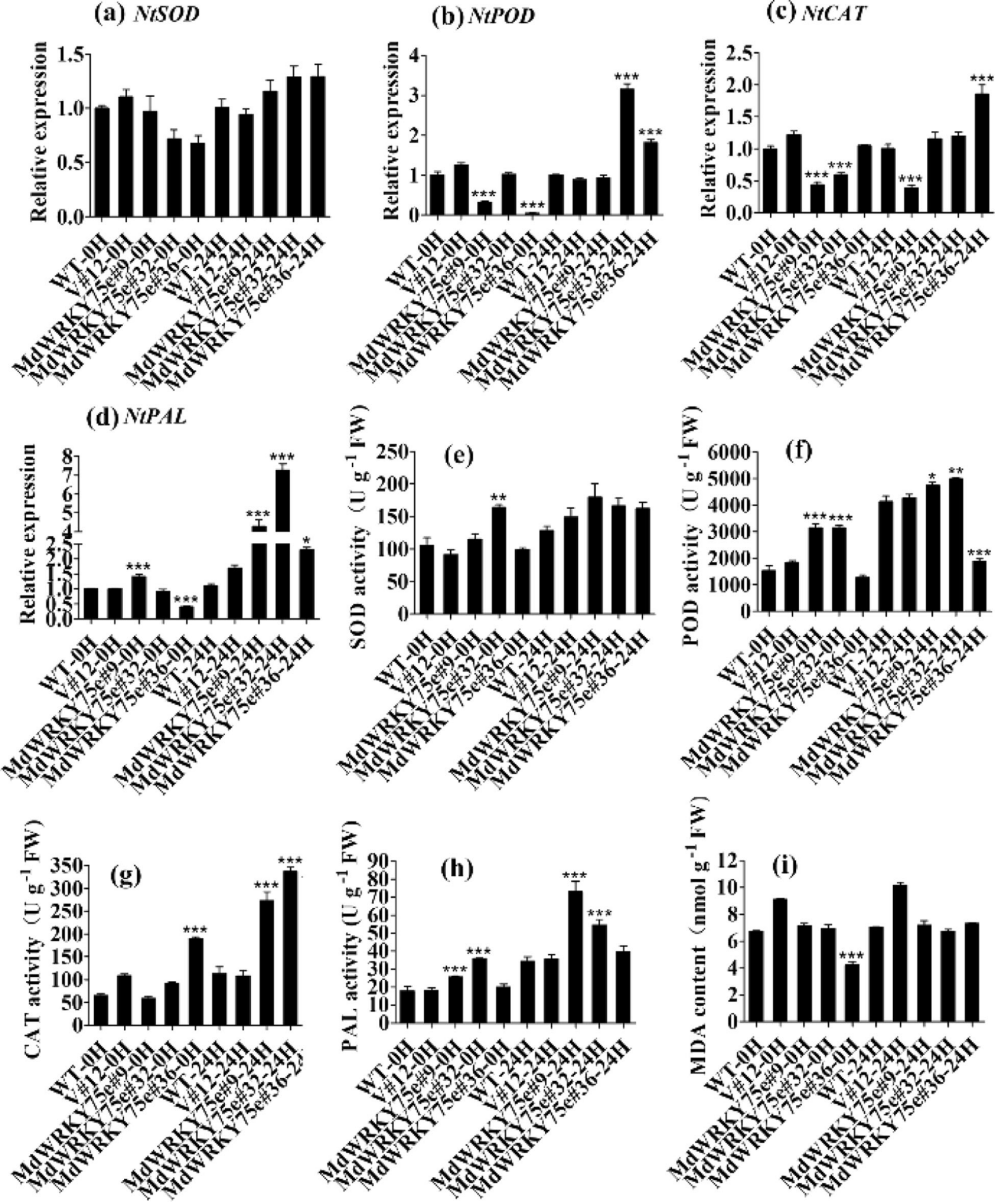
Analysis of antioxidant enzyme genes and antioxidant enzyme activities and MDA content in tobacco. **a**–**d** Expression of *NtSOD* (**a**), *NtPOD* (**b**), *NtCAT* (**c**), and *NtPAL* (**d**) in WT, V#12, and *MdWRKY75e* transgenic lines (#9, #32, and #36) at 0 and 24 h of *B. cinerea* infection. **e**–**i** Activity of SOD (**e**), POD (**f**), CAT (**g**), PAL (**h**), and MDA content (**i**) in WT, V#12, and *MdWRKY75e* transgenic lines at 0 and 24 h of *B. cinerea* infection. WT: wild-type; V#12: empty vector; and *MdWRKY75e*#9, *MdWRKY75e*#32, and *MdWRKY75e*#36: *MdWRKY75e* transgenic lines

**Fig. 9:**
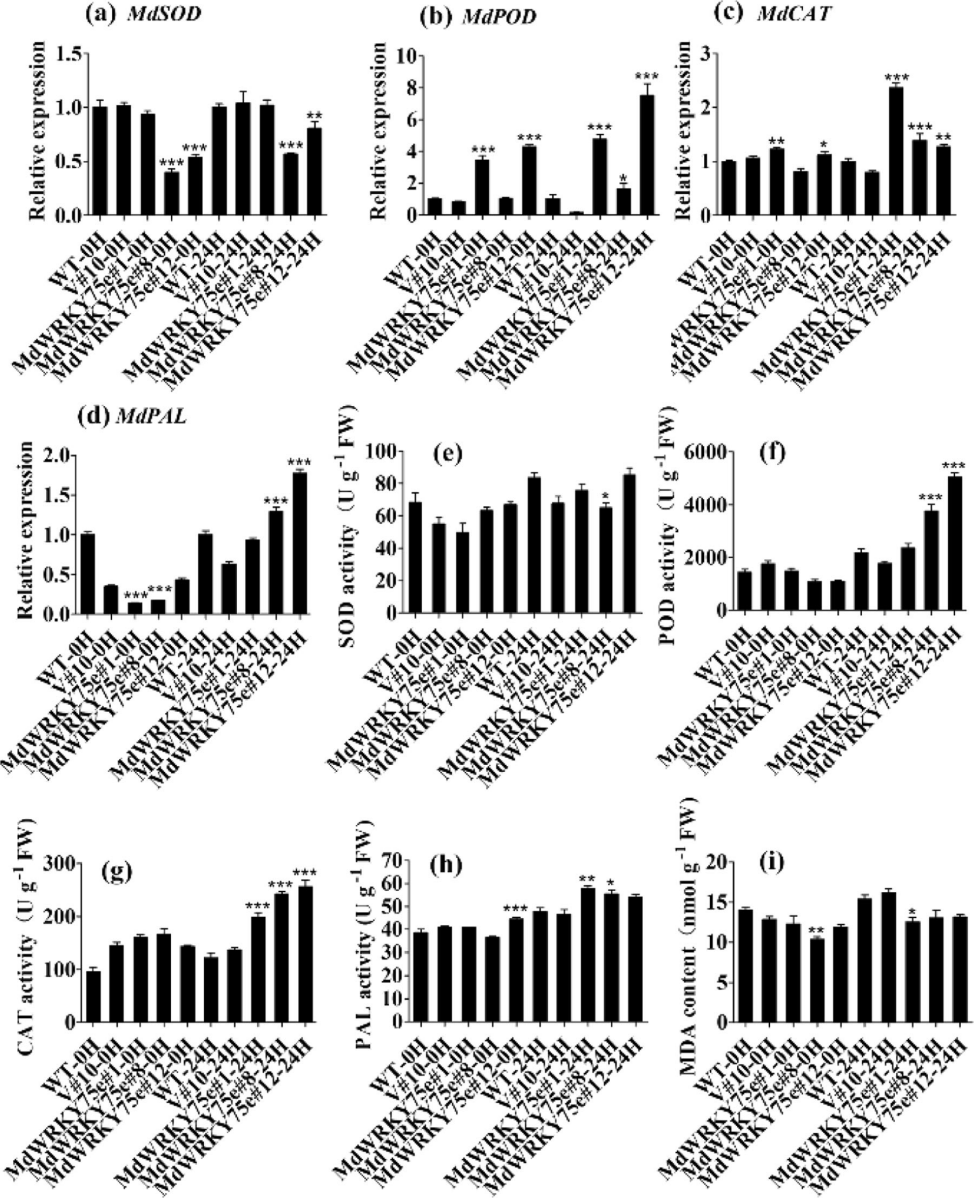
Analysis of antioxidant enzyme genes and antioxidant enzyme activities and MDA content in apple. **a**–**d** Expression of *MdSOD* (**a**), *MdPOD* (**b**), *MdCAT* (**c**), and *MdPAL* (**d**) in WT, V#10, and *MdWRKY75e* transgenic lines (#1, #8, and #12) at 0 and 24 h of *A. alternata* infection. **e**–**i** Activity of SOD (**e**), POD (**f**), CAT (**g**), PAL (**h**), and MDA content (**i**) in WT, V#10, and *MdWRKY75e* transgenic lines at 0 and 24 h of *A. alternata* infection. WT: wild-type; V#12: empty vector; and *MdWRKY75e*#1, *MdWRKY75e*#8, and *MdWRKY75e*#12: *MdWRKY75e* transgenic lines

Furthermore, we examined the activities of four enzymes, SOD, CAT, POD, and PAL, as well as the MDA content in WT, empty vector, and transgenic lines after infection with pathogens. Interestingly, the enzyme activities of POD, CAT, and PAL were slightly higher in GL-3 transgenic lines than in WT and empty vector lines under normal conditions. Infection with pathogens significantly increased the activities of these enzymes in transgenic plants compared to those in WT and empty vector plants. In contrast, the enzyme activity of SOD and the MDA content in GL-3 transgenic lines under normal conditions were not significantly different from those after infection with pathogens, consistent with the results obtained in transgenic tobacco. Furthermore, we examined the expression of *NtSOD/MdSOD*, *NtCAT/MdCAT*, *NtPOD/MdPOD*, and *NtPAL/MdPAL* in tobacco and GL-3 transgenic lines and found that except for *NtSOD/MdSOD*, the transcription levels of the other genes were significantly higher in the transgenic plants, both before and after infection with pathogens; however, these transcription levels did not change remarkably between WT and empty vectors before and after pathogen infection treatment. Notably, these transcription levels were remarkably higher in the transgenic plants than in the WT and empty vector plants (Figs. 8 and 9).

### Expression of genes involved in disease resistance responses in WT, empty vector, and transgenic lines before and after pathogen infection

**Fig. 10:**
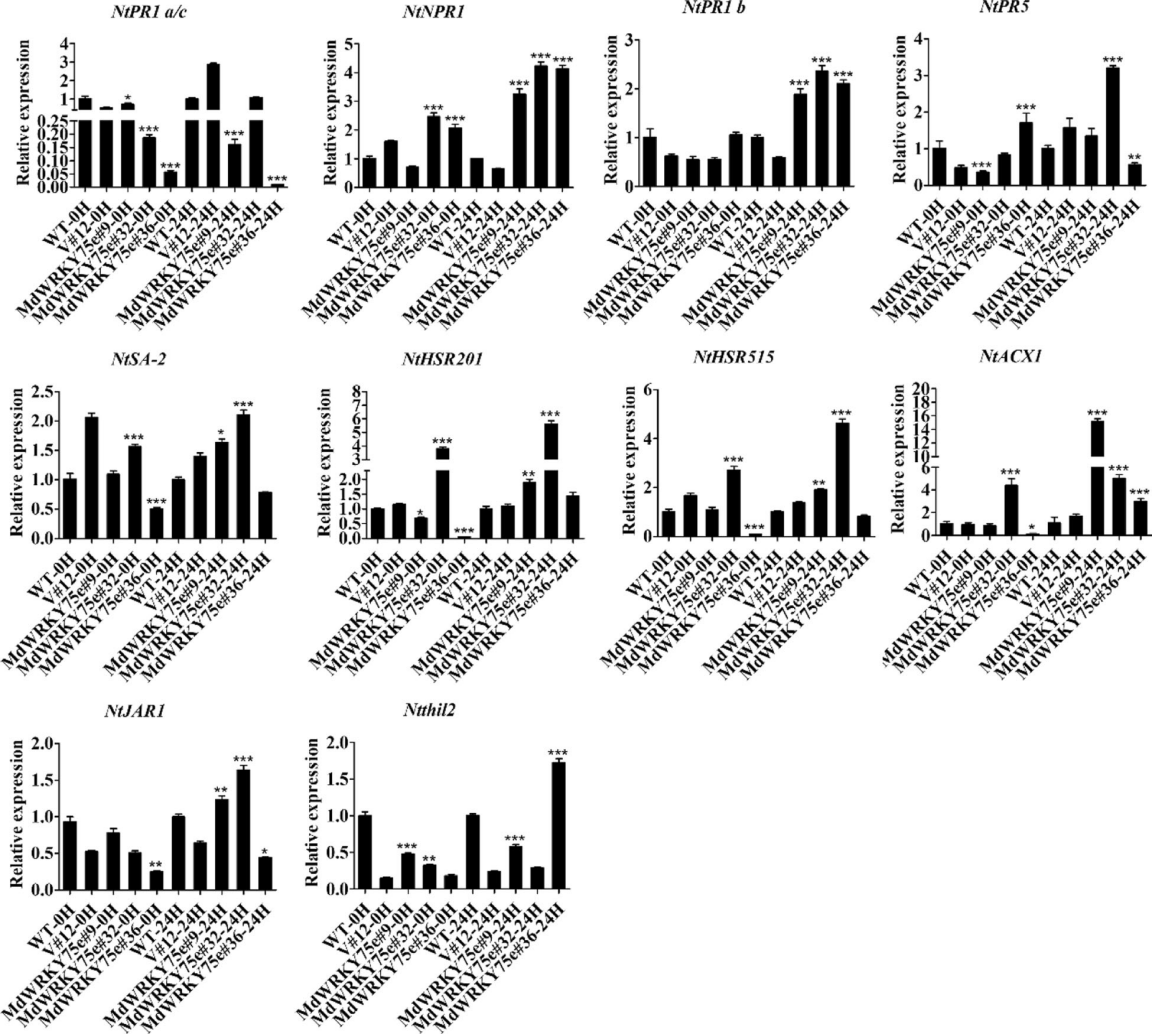
Expression profiles of the eleven disease resistance-responsive genes in wild-type (WT), empty vector (V#12), and *MdWRKY75e* transgenic tobacco lines (#9, #32, and #36) at 0 and 24 h of *B. cinerea* infection. WT: wild-type; V#12: empty vector; and *MdWRKY75e*#9, *MdWRKY75e*#32, and *MdWRKY75e*#36: *MdWRKY75e* transgenic lines

**Fig. 11:**
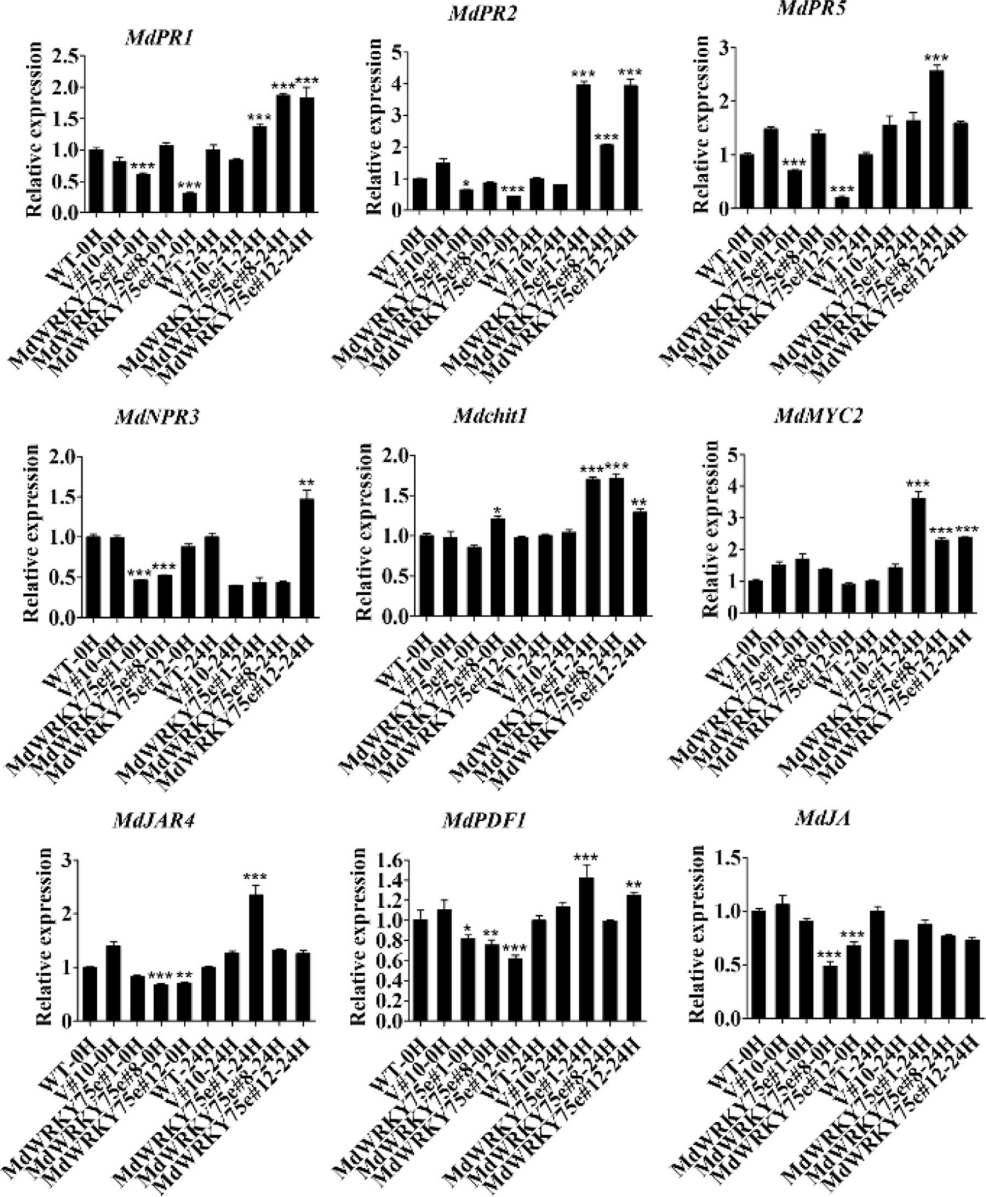
Expression profiles of the nine disease resistance-responsive genes in wild-type (WT), empty vector (V#10), and *MdWRKY75e* transgenic apple lines (#1, #8, and #12) at 0 and 24 h of *A. alternata* infection. WT: wild-type; V#10: empty vector; and *MdWRKY75e*#1, *MdWRKY75e*#8, and *MdWRKY75e*#12: *MdWRKY75e* transgenic lines

The expression patterns of some genes involved in disease resistance responses were analyzed by qRT–PCR. Under normal conditions, the expression levels of the genes did not change in transgenic tobacco lines (#9, #32, and #36) and GL-3 transgenic lines (#1, #8, and #12) compared to those in the WT and empty vector lines. The levels of *NtPR5*, *NtHSR201*, *NtHSR515*, *NtACX1*, *NtJAR1*, and *Ntthil2* transcripts were slightly higher in the transgenic lines than in the WT and empty vector lines after infection with pathogens. In particular, *NtNPR1* and *NtPR1b* expression was remarkably increased in the transgenic line compared to that in the WT and empty vector lines. However, the expression levels of the *NtPR1 a/c* and *NtSA-2* genes in the transgenic plants were not notably different in comparison to those in the WT and empty vector plants (Fig. 10). Similarly, the *MdPR1*, *MdPR2*, *Mdchit1*, *MdMYC2*, and *MdPDF1* transcript levels were particularly higher in the transgenic lines than in the WT and empty vector lines after infection with pathogens. Additionally, the expression levels of *MdPR5*, *MdJAR4*, and *MdJA* were slightly higher in the transgenic plants than in the WT and empty vector plants. Nevertheless, the level of *MdNPR3* transcripts in the transgenic plants did not change compared to that in the WT and empty vector plants (Fig. 11).

### MdWRKY75e directly interacts with the promoter of *MdLAC7*

**Fig. 12:**
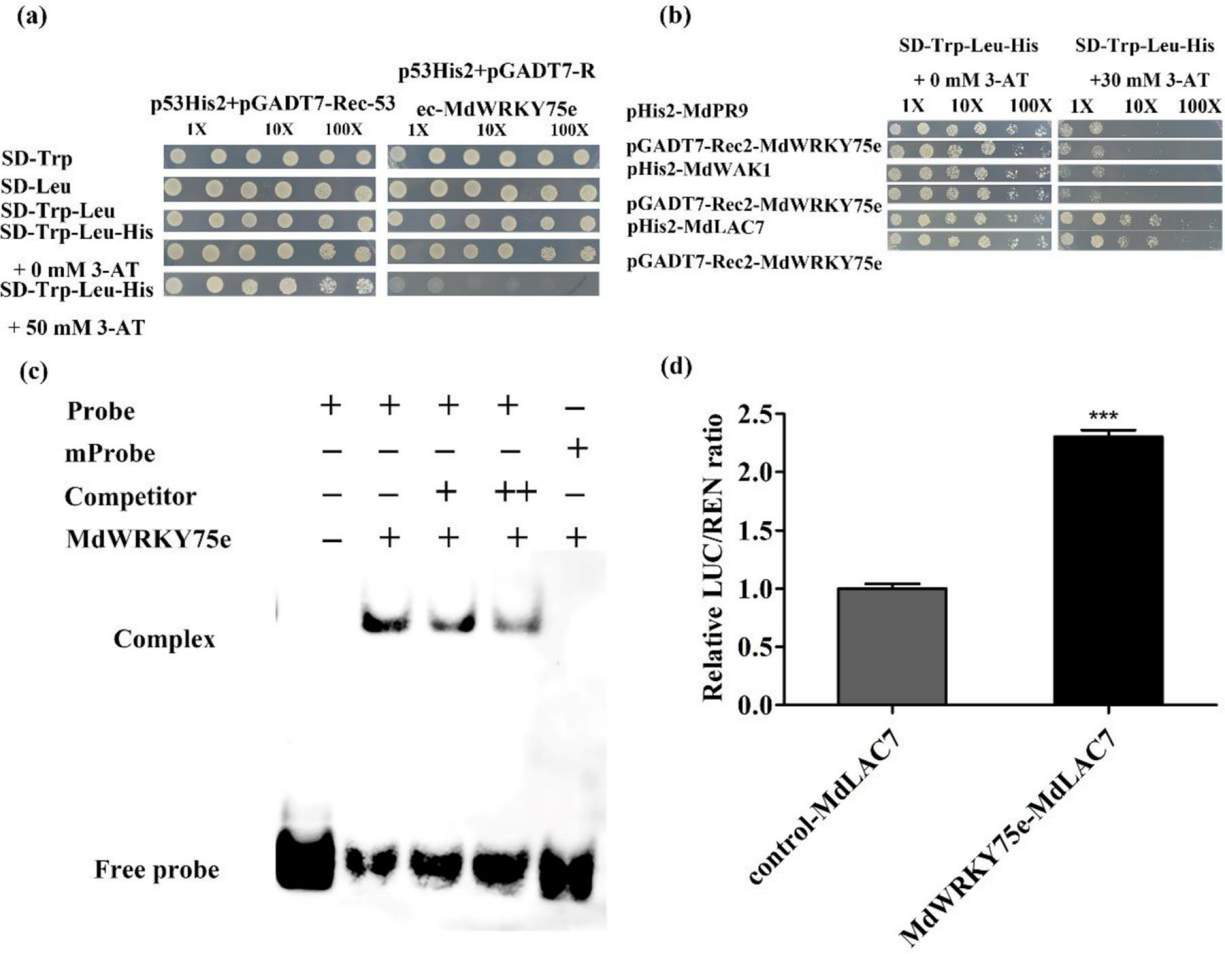
MdWRKY75e associates with the promoter of *MdLAC7* and initiates its expression. **a** Positive and negative constructs were utilized for the yeast one-hybrid experiment. **b** Prey and bait vectors were utilized for the yeast one-hybrid test. **c** The MdWRKY75e-MBP fusion protein directly bound to the W-box elements of the MdLAC7 promoters. – absence, + presence. **d** Real-time expression experiment of the activity of promoters coconverted with the MdWRKY75e effector and MdLAC7 reporter in tobacco leaves

Bioinformatics analysis indicated that the promoter sequences of *MdLAC7*, *MdWRK1*, *MdFRK1*, and *MdPR9*, with sizes of 1221, 1318, 1977, and 1530 bp, respectively, contained potential W-box (TTGACC) elements in their upstream regions (Supplementary Table S3). The upstream transcription start sites of the promoter sequences of *MdLAC7*, *MdWRK1*, *MdFRK1*, and *MdPR9* were cloned into the reporter vector pHIS2 as bait, while MdWRKY75e was used as prey (Supplementary Fig. S8a). First, self-activating reporter vectors were filtered by the concentration of 3-AT, which only inhibited pHIS2-LAC7-, MdWRK1-, and MdPR9-transformed Y187 yeast cell growth at 30 mM. However, pHIS2-MdFRK1 self-activation was not inhibited by 100 mM 3-AT (Supplementary Fig. S8b). The vectors cotransformed with bait (pHIS2-LAC7, MdWRK1, and MdPR9) and prey (MdWRKY75e) and negative control yeast cells grew normally in the selection medium. However, the growth of yeast cells in the negative control was completely suppressed by the addition of 30 mM 3-AT, while that in the positive control and bait (pHIS2-LAC7) and prey (MdWRKY75e) vector-containing cells were not (Fig. 12a, b). EMSA showed that the formation of a protein–DNA complex was observed when the MdWRKY75e-MBP fusion protein was incubated with the probe containing the W-box element, whereas the complex was inhibited by the competitor probe. In addition, mutation of the W-box element in the probe was completely abolished (Fig. 12c). These results suggest that MdWRKY75e activates *MdLAC7* promoter transcription. Transient expression tests were conducted for the interaction between MdWRKY75e and the promoter of *MdLAC7*. Transient expression experiments for transforming the LUC reporter gene into tobacco leaves indicated that the LUC/REN ratio was considerably higher in leaves transformed with effectors and reporters than in controls (Supplementary Fig. S8c, d and Fig. 12d). Taken together, MdWRKY75e serves as a transactivator of MdLAC7.

## Discussion

Plant WRKY proteins reportedly play a pivotal role in physiological and biological processes, such as hormonal signaling, carbohydrate synthesis, senescence, development, and secondary metabolite synthesis^26,41^. The WRKY TF superfamily consists of 72 and 119 members in *Arabidopsis* (*Arabidopsis thaliana*) and apple (*M. domestica*), respectively^42,43^. *A. thaliana AtWRKY75* and *Fragaria ananassa Fa WRKY1* (*AtWRKY75* homologous gene) play a pivotal role in modulating biological stress responses^27,29,44^. Conversely, *FaWRKY1* negatively regulates resistance to the phytopathogenic fungus *Colletotrichum acutatum* in strawberry fruit^45^. Herein, we identified the WRKY TF *MdWRKY75e* (Group IIc) in *M. domestica* and demonstrated that its overexpression enhanced tolerance to infection with pathogens. We further showed that MdWRKY75e is a positive regulator of *MdLAC7* expression as well as laccase and lignin biosynthesis. Therefore, this study uncovered the molecular mechanism of MdWRKY75e and connected the function of WRKY to laccase and lignin biosynthesis to strengthen disease resistance.

Overexpression of WRKY genes in model or nonmodel plants reportedly induces tolerance to biotic stresses^27–29,33,34,44,46,47^, suggesting that WRKY genes may have a high potential for disease resistance. Here, *MdWRKY75d* and *MdWRKY75e* transcript levels were markedly increased by *A. alternata* infection. Analysis of the tissue-specific expression of *MdWRKY75d* and *MdWRKY75e* showed that these genes were mainly involved in fruit development and leaf development, respectively. Transgenic lines overexpressing *MdWRKY75e* displayed better disease resistance to infection with pathogens such as *B. cinerea* and *A. alternata*. However, *MdWRKY75d* transgenic lines displayed poor resistance to infection with pathogens such as *B. cinerea*. *MdWRKY75e* knockdown rendered apple slightly susceptible to *A. alternata*, indicating that MdWRKY75e is a positive regulator of disease resistance.

ROS accumulation in biological systems relies on the homeostasis between the formation and removal of ROS under physiological conditions^19,20^. We evaluated ROS levels in transgenic and WT apples. Histochemical staining with NBT and DAB revealed lower contents of H_2_O_2_ and O_2_^−^ in the transgenic lines than in the WT and empty vector lines after infection with pathogens. Plant cells have a sophisticated antioxidant defensive system for ROS removal, which is accomplished by ROS scavenging enzymes, such as POD, CAT, and SOD^20^. The activities of CAT and POD were observed to be much higher in transgenic tobacco and GL-3 plants than in their respective WT and empty vector lines after infection with pathogens. However, SOD activities and the MDA content did not change in tobacco and GL-3 transgenic plants. These genes are also involved in the biosynthesis of antioxidant enzymes (*NtSOD/MdSOD*, *NtCAT/MdCAT*, and *NtPOD/MdPOD*). qRT–PCR analysis revealed that the expression levels of these genes, except for *NtSOD/MdSOD*, were higher in the transgenic plants than in the WT and empty vector plants after infection with pathogens, indicating that *MdWRKY75e* overexpression effectively induces a detoxification system to remove ROS generated during infection with pathogens.

The pathogenesis-related genes *PR-1* and *PR-2* (β-1,3-glucanase), the SA-responsive marker gene *NPR1*, and *PR-5* (thaumatin-like proteins) are often activated by SA to mediate plant defense responses^48,49^. Similarly, *MYC2*, *JAZ*, *Chit1,* and the JA-responsive fungal pathogen marker genes *Thi2.1* and *PDF1.2* are often activated by JA to mediate plant defense responses^17,18,50,51^. Liu et al.^52^ found that *NtPR1a* overexpression may be mainly dependent on the hypersensitive response (HR)-associated genes *NtHSR201*, *NtHSR515*, and *NtHIN1*; the SA-associated genes *NtPR1* and *NtPR2*; and the JA-associated gene *NtPR1b* to enhance resistance to *R. solanacearum* in tobacco. Our results showed that *NtNPR1*, *NtPR1b*, *MdPR1*, *MdPR2*, *Mdchit1*, *MdMYC2*, and *MdPDF1* transcript levels were remarkably upregulated in transgenic plants compared with those in WT and empty vector plants. *NtPR5*, *NtHSR201*, *NtHSR515*, *NtACX1*, *NtJAR1*, *Ntthil2*, *MdPR5*, *MdJAR4*, and *MdJA* transcript levels were slightly higher in transgenic plants than in WT and empty vector plants, both before and after infection with pathogens. However, *NtPR1 a/c*, *NtSA-2*, and *MdNPR3* expression levels hardly changed in the transgenic plants in comparison to those in the WT and empty vector plants (Figs. 10 and 11). More importantly, *WRKY62* and *WRKY70* play an essential role in the crosstalk between the SA and JA pathways, acting as regulatory nodes of the JA and SA signaling pathways^53,54^. *MdWRKY75e* transcript levels were markedly increased by MeJA treatment but suppressed by SA treatment. Furthermore, an investigation of endogenous hormones showed that JA and methyl jasmonate contents were higher in transgenic lines than in WT and empty vector lines. SA and methyl salicylate levels were remarkably lower in transgenic lines than in WT and empty vector lines. These results demonstrated that JA signaling pathways play key roles in the enhanced disease resistance of transgenic plants overexpressing *MdWRKY75e*, while SA signaling pathways play secondary roles in this regard.

PAL, peroxidases, and laccases have important roles in lignin synthesis in the phenylpropanoid pathway, which provides plants with a physical barrier against pathogens and pests^22–24,55,56^. Interestingly, the activities of PAL, POD, and laccases were significantly higher in transgenic plants than in WT and empty vector plants. Leaf and root dry weights were higher in transgenic plants than in WT and empty vector plants. The roots of transgenic MdWRKY75e plants had deeper color and increased lignification, and root hair formation was particularly suppressed in GL-3 transgenic lines; this was similar to observations of AtWRKY75^57,58^. Notably, the lignin contents in the leaves and roots of MdWRKY75e transgenic plants were markedly higher than those of WT and empty vector plants. However, the laccase activity and lignin content were not significantly different, although they were slightly lower in transgenic lines than in the WT, empty vector (pTRV2), and pTRV2-*GFP* lines. *MdWRKY9* overexpression conferred intensive dwarfing in the M26 rootstock of apple^59^. We further observed that three GL-3 transgenic lines (#1, #8, and #12) exhibited dwarfism, uniform serration of the leaf edges, and a greener leaf color after 2 months of transplanting; these phenotypes indicate enhanced stress resistance.

**Fig. 13:**
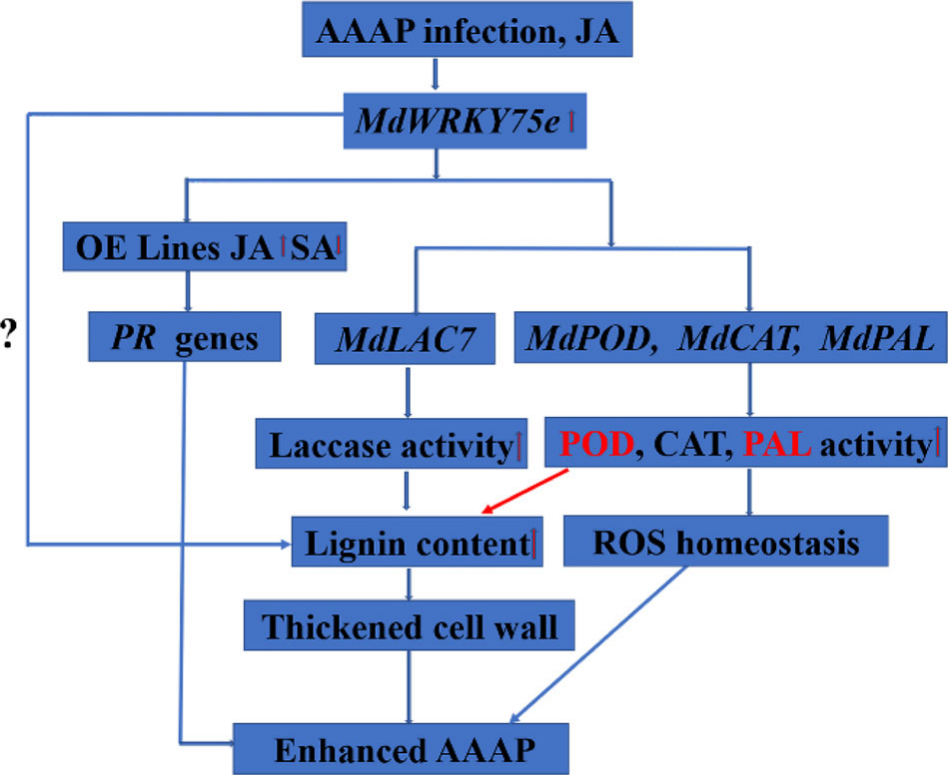
A suggested model of the modulatory function of *MdWRKY75e* in response to AAAP infection. The JA signaling pathway is activated by AAAP (*A. alternata* apple pathotype) infection, resulting in increased expression levels of *MdWRKY75e*, which in turn modulates *MdLAC7* by binding to W-box elements in its promoter. Upregulation of the MdWRKY75e–MdLAC7 interaction boosts laccase and lignin synthesis to strengthen mechanical defense capabilities, allowing AAAP infection-associated damage to be mitigated by sustaining ROS equilibrium and PR gene regulation

At the transcriptional level, WRKY TFs can specifically regulate target gene expression by binding to the W-box (T)TGACC(A/T) in their promoters^41^. We examined the W-box elements of *MdLAC7* (laccase 7), *MdWRK1*, *MdFRK1*, and *MdPR9* (POD). Interestingly, *MdLAC7*, *MdWRK1*, *MdFRK1*, and *MdPR9* expression (especially *MdLAC7*) was intensely induced in *MdWRKY75e*-overexpressing GL-3 lines after infection with pathogens. Yeast one-hybrid, EMSA, and transient expression experiments supported the straightforward and particular interactions between MdWRKY75e and the *MdLAC7* promoter. These data enabled us to speculate that *MdLAC7* is a target gene of MdWRKY75e, indicating that MdLAC7 may be regulated by WRKY TFs. Here, we present a model of the role of the modulatory function of *MdWRKY75e* during the response to infection with pathogens, which may directly regulate the synthesis of lignin (Fig. 13). Infection with pathogens induces the JA signaling pathway, resulting in elevated expression of *MdWRKY75e*, which regulates *MdLAC7* by binding to W-box elements in its promoter. Upregulation of the MdWRKY75e‒MdLAC7 interaction contributes to laccase and lignin synthesis, and ROS scavenging systems contribute to maintaining ROS homeostasis. We provide herein a new understanding of the molecular mechanisms of laccase and lignin synthesis and ROS removal in disease resistance.

In summary, we confirmed a disease resistance-responsive WRKY TF, MdWRKY75e, extracted from ‘Sushuai’ apple that serves as an active modulator of disease resistance. *MdWRKY75e*-overexpressing plants accumulated more laccase and lignin, which in turn stimulated downstream *MdLAC7* expression. Establishment of the WRKY-LAC7 and ROS scavenging network offers useful knowledge of the function and potential molecular mechanism of WRKY and broadens our understanding of the complicated disease resistance signaling network. However, *MdWRKY75e* may also regulate other disease resistance-responsive genes, and further studies are needed to uncover other components associated with *MdWRKY75e* to gain deeper insight into the molecular mechanisms of *MdWRKY75e* function in disease resistance.

## Materials and methods

### Plant material, growth environment, and stress treatments

*Nicotiana tabacum* (K326), ‘Sushuai’ apple exhibiting greater disease resistance^60^, and transgenic (tobacco and GL-3) plants overexpressing *MdWRKY75d* and *MdWRKY75e* derived from ‘Sushuai’ apple were cultivated in Nanjing, China, under normal farming practices or grown in a greenhouse. Leaves were excised from *M. domestica* ‘Sushuai’ apple and transgenic lines (tobacco and GL-3) for infection with pathogens as well as 0.1 mM JA and 0.1 mM SA treatments. The leaves were cleaned and cultivated for 6 h to reduce mechanical damage, followed by exposure to appropriate treatments. *N. tabacum* (K326) and *B. cinerea* strain Bt56 preserved in the laboratory were used. All biological samples were stored at −80 °C until further analysis.

### RNA extraction and quantitative real-time PCR analysis

Total RNA was derived from frozen leaves using an RNA extraction kit (Fuji, China) and treated with RNase-free DNase I (TaKaRa, Tokyo, Japan) to reduce potential redundant genomic DNA. First-strand cDNA was generated using a reverse transcription kit (TaKaRa, Tokyo, Japan) following the manufacturer’s instructions. qRT–PCR was conducted with a SYBR-Green PCR kit (TakaRa, Tokyo, Japan) according to the manufacturer’s instructions. Each sample was analyzed in four replicates, and the 2^−ΔΔCt^ method^61^ was used to compute the relative expression levels of each gene. *Tubulin* was used as the reference gene for *M. domestica* and tobacco to normalize gene expression levels. The primers were designed using primer-BLAST (https://www.ncbi.nlm.nih.gov/tools/primer-blast) (Supplementary Table S1).

### *MdWRKY75s* cloning and sequence analysis

The *MdWRKY75* sequence (https://phytozome-next.jgi.doe.gov/info/Mdomestica_v1_1) was used as a template for designing gene-specific primers (Supplementary Table S2) to amplify *MdWRKY75d* and *MdWRKY75e* genes using RT–PCR. The PCR product was purified, subcloned into the clone007 blunt simple vector, and sequenced at TsingKe (Nanjing, China). Multiple alignments of the homologous amino acid sequence of MdWRKY75s from different species from the NCBI website were performed using Clustal W and BioXM. The phylogenetic tree was created in MEGA 7.0 software by the neighbor-joining method and bootstrap test using 1000 replicates. The molecular weight and theoretical isoelectric point were forecasted using the Expert Protein Analysis System (http://web.expasy.org/compute_pi/).

### Subcellular localization and transactivating activity of MdWRKY75s

PCR amplification of the full-length cDNAs of *MdWRKY75d* and *MdWRKY75e* was conducted using primers (Supplementary Table S2) comprising *Nco*I and *Spe*I restriction sites, and then the cDNAs were ligated to the pCAMBIA1302 vector to produce a fusion construct (*35S-MdWRKY75-GFP*). After sequence identification, the control vector (pCAMBIA1302) and the fusion construct (35S-*MdWRKY75-GFP*) were transformed into *Agrobacterium tumefaciens* strain AH105 by heat shock. The 35S-*MdWRKY75-GFP* and free 35S-*GFP* plasmids were transiently coexpressed with a nuclear marker (35S-*D53-RFP* vector)^62^.

For the transactivation assay, the CDSs of *MdWRKY75d* and *MdWRKY75e* were amplified by PCR using primers (Supplementary Table S2) comprising *Nco*I and *Bam*HI restriction sites. The resultant fragments containing MdWRKY75d and MdWRKY75e were fused via recombination reactions downstream of the yeast GAL4 DNA binding domain of pGBKT7. The negative control (pGBKT7) and the fusion vector were separately expressed following the manufacturer’s instructions in yeast strain AH109.

### Vector construction, plant transformation, and identification of transgenic lines

The full-length coding regions of *MdWRKY75d* and *MdWRKY75e* were PCR-amplified and (Supplementary Table S2) inserted into the pCAMBIA1301 vector. The leaf disc transformation method was used to transform tobacco K326^63^. Normal growth of transgenic tobacco was identified by cutting the leaves of resistant tobacco for GUS staining, followed by semiquantitative RT–PCR analysis and qRT–PCR of the gene-specific primers^64^.

The full-length coding region of *MdWRKY75e* was PCR-amplified (Supplementary Table S2) and inserted into the pCAMBIA2300 vector. Transgenic GL-3 apple plants were produced from leaf fragments by *Agrobacterium*‐mediated transformation^65^. We performed GUS staining and semiquantitative RT–PCR analysis with isolated RNA to check for the presence of the transgene in putatively transformed lines. Overexpression of *MWRKY75e* was confirmed by qRT–PCR.

### Assessment of pathogen tolerance in transgenic lines

The WT, empty vector, and transgenic lines were infected with pathogens to investigate their disease resistance capacities. For pathogen infection treatment, leaves were collected from 45-days-old seedlings of tobacco transgenic lines and 60-days-old seedlings of apple transgenic lines. The plant pathogens *B. cinerea* and *A. alternata* were grown on potato dextrose agar (PDA) media (Solarbio, Beijing, China) at 24 °C for 8 days. Fifteen to twenty leaves of the WT, empty vector, and transgenic lines (tobacco and apple) were collected, washed with ddH_2_O three times, and cultured for 6 h for infection with pathogens. The leaves were infected using an approximately 3-mm diameter bacterial plaque acquired by a hole puncher to maintain consistency. Afterward, images were taken at various time points, and the leaf lesion areas were measured by ImageJ software^65,66^.

Samples of leaves and roots corresponding to the various time points were collected and frozen instantly in liquid nitrogen and stored at −80 °C for analyses of gene expression and enzyme activity. The MDA content, antioxidant enzyme activities, laccase activity, and lignin content in the leaves, and partial index in the roots at the corresponding time points were examined. Infection with pathogens was repeated at least three times. Three replicates were utilized for each line after the infection treatment.

### Promoter isolation and Y1H assay

Genomic DNA was extracted from apple leaves by applying a DNA extraction kit (TIANGEN). PCR amplification of the promoter sequences of *MdPR9*, *MdRFK1*, *MdWAK1*, and *MdLAC7* was accomplished with primers (Supplementary Table S2) using genomic DNA as a template. The amplified products were subsequently subcloned into the clone007 blunt simple vector and then sequenced at TsingKe. Transcription start sites and conserved cis-element motifs of promoters were analyzed using the PLACE (http://www.dna.affrc.go.jp/htdocs/PLACE/) and Plant-CARE (http://bioinformatics.psb.ugent.be/webtools/plantcare/html/) websites, respectively. For this purpose, full-length *MdWRKY75e* was RT–PCR-amplified using primers (Supplementary Table S2) and incorporated into the *Eco*RI and *Xho*I sites of pGADT7-Rec to produce the effector vector pGADT7-Rec-*MdWRKY75e*. Simultaneously, promoter sequences of *MdPR9*, *MdRFK1*, *MdWAK1*, and *MdLAC7* were amplified using primers (Supplementary Table S2) comprising the *Eco*RI and *Sac*I restriction sites and subcloned into pHis2 to generate the reporter vectors pHis2-MdPR9, pHis2-MdRFK1, pHis2-MdWAK1, and pHis2-MdLAC7, respectively. A yeast one-hybrid experiment was conducted using the Matchmaker Gold Yeast One-Hybrid Library Screening System (Clontech, USA). Y187 yeast cells cotransformed with effector vector and reporter vector were cultured at 28 °C for 3 days on SD-Trp-Leu-His medium supplemented with or without 30 mM 3-AT.

### Electrophoretic mobility shift assay

Electrophoretic mobility shift assay (EMSA) was performed using an EMSA Probe Biotin Labeling Kit and a Chemiluminescent EMSA Kit (Beyotime Biotechnology, Shanghai, China). The CDS of the *MdWRKY75e* gene was cloned into the pMAL-c5X vector, which was then transformed into Rosetta (DE3) cells for the subsequent production of the MdWRKY75e-MBP fusion protein. Probes specific for the promoter fragments and their mutants (Supplementary Table S2) were synthesized by Sangon Biotechnology Co., Ltd. (Shanghai, China). In *MdLAC7*, the 5′-TTGACC-3′ motif was replaced by 5′-TCGAAC-3′ in the mutant probe. EMSAs were performed according to the manufacturer’s protocol and as described^67^.

### Transient expression assay

VIGS-mediated inhibition of *MdWRKY75e* was conducted as described previously^68,69^. To construct the pTRV2-*MdWRKY75e* vector, a 180 bp fragment of the *MdWRKY75e* CDS (24–204 bp) was inserted into the *EcoR*I and *Xho*I sites of tobacco rattle virus-based vector 2 (pTRV2) to produce the pTRV2-*MdWRKY75e* vector. Empty pTRV2 and pTRV2-*GFP* constructs were utilized as controls.

Primers containing *Sac*I and *Bam*HI restriction sites were used to amplify the coding region of *MdWRKY75e* (Supplementary Table S2), which was inserted into pCAMBIA1301 to yield an effector plasmid. *MdPR9*, *MdRFK1*, *MdWAK1*, and *MdLAC7* promoter sequences were amplified with specific primers (Supplementary Table S2) containing *Hind*III and *Bam*HI restriction sites and inserted into the pGreen II 0800-LUC reporter vector^70^. The effector and reporter vectors were transfected into *A. tumefaciens* GV3101 cells. Transient expression experiments were conducted in tobacco as described previously^71,72^ with minor modifications. The analysis of the LUC/REN ratio was performed as described previously^73^.

### Histochemical staining and physiological measurement

GUS staining was conducted as described previously^31^. Histochemical staining for H_2_O_2_ and O_2_^−^ accumulation was carried out with DAB and NBT, respectively^74,75^. H_2_O_2_ and O_2_^−^ levels were measured using analytical kits (Lai Er Bio-Tech, HeFei, China). Toluidine blue O staining of paraffin-embedded sections was performed as described previously^65,76^.

Analysis of the MDA content, SOD, POD, CAT, and PAL enzyme activities, laccase activity, and lignin content levels was performed using analytical kits (Lai Er Bio-Tech, HeFei, China).

### Measurement of SA and JA contents

Quantitative measurement of SA and JA contents was performed using an Agilent 1290/AB Qtrap6500 HPLC–MS/MS Analysis system (Agilent, Santa Clara, CA, USA) following the manufacturer’s protocol, as described previously^77,78^.

### Statistical analysis

The experiments were repeated at least three times for each stress treatment. For a representative experiment with at least three independent replicates, data are expressed as the mean ± SE. Data were analyzed by Tukey’s multiple range tests in the ANOVA program of SPSS (IBM SPSS 22, Chicago, IL, USA). *P < 0.05, **P < 0.01, and ***P < 0.001 were considered significant.

## Supplementary information

*MdWRKY75e* enhances resistance to *Alternaria alternata* in *Malus domestica*

Supporting Information

Supporting Information

## Data Availability

The data used to support the findings of this study are available from the corresponding author upon reasonable request.
