# Supplementary material for: MdWRKY75e enhances resistance to Alternaria alternata in Malus domestica
Source: Hortic Res. 2021 Oct 11;8:225. doi: 10.1038/s41438-021-00701-0 (PMC8502781; doi:10.1038/s41438-021-00701-0)
Supplement: Supplementary file 2 — Supporting Information [file 41438_2021_701_MOESM2_ESM.docx]

## Supporting Information


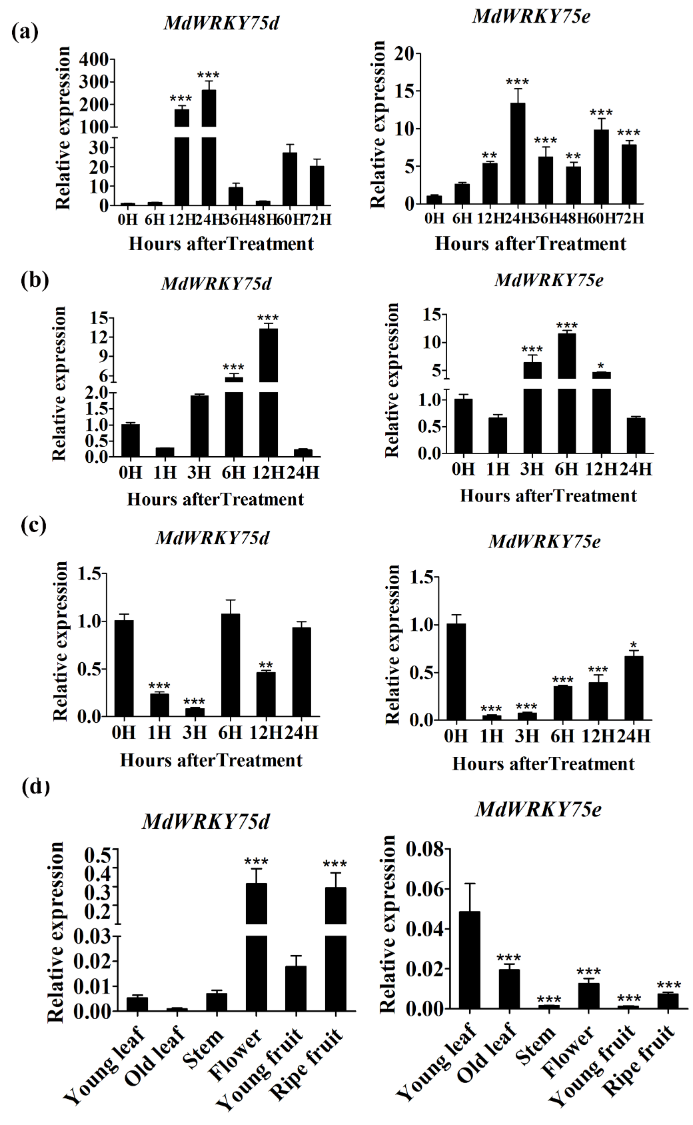


**Fig. S1** Time-course of the expression of *MdWRKY75d* and *MdWRKY75e* under the *A. alternata*, MeJA, SA, and different organs in ‘Sushuai’ apple. (a-c) Time-course of *MdWRKY75d* and *MdWRKY75e* expression levels under the *A. alternata*, 0.1 mM MeJA, and SA in ‘Sushuai’ apple. (d) Expression patterns of *MdWRKY75d* and *MdWRKY75e* during vegetative growth in the young leaves, old leaves, stems, flowers, young fruits, and old fruits. For the treatments, the expression level at 0 h was set as 1.0 and data represented means ± SE of three replicates. *Tubulin* expression in apple was used as an internal control. The differences were analyzed by Tukey’s multiple range test. Different stars indicate significantly different (**P* < 0.05; ***P* < 0.01; ****P* < 0.001).


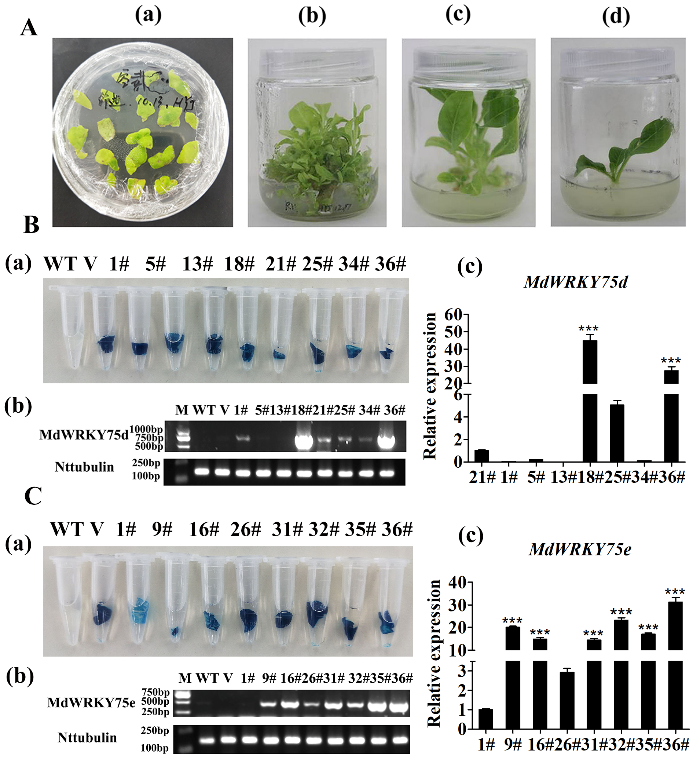


**Fig. S2** Generation and molecular identification of transgenic tobacco plants overexpressing *MdWRKY75d* and *MdWRKY75e*. (A) Genetic transformation process of the tobacco leaf discs via *Agrobacterium*-mediated transformation of the overexpression vector (a-d). (B) GUS staining (a), semi-quantitative RT-PCR analysis (b), and expression of *MdWRKY75d* by qRT-PCR (c) in the wild type (WT), empty vector (V), and transgenic lines. (C) GUS staining (a), semi-quantitative RT-PCR analysis (b), and expression of MdWRKY75e by qRT-PCR (c) in the wild type (WT), empty vector (V), and transgenic lines. M, DNA marker. WT, wild type. V, empty vector. The numbers and # indicated different transgenic lines. The differences were analyzed by Tukey’s multiple range test. Different stars indicate significantly different (**P* < 0.05; ***P* < 0.01; ****P* <
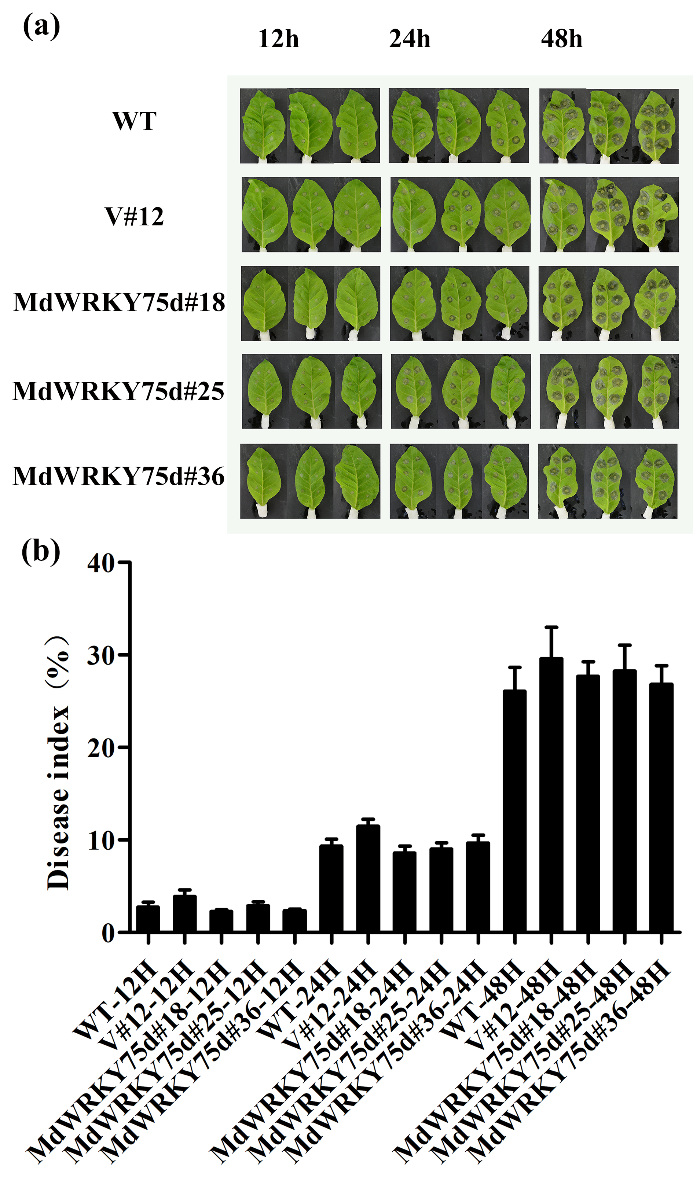
0.001).

**Fig. S3** Overexpression of *MdWRKY75d* *B. cinerea* infection phenotypes in transgenic tobacco. (a) Time-course of *B. cinerea* infection of WT, V#12, and *MdWRKY75e* transgenic lines for 48 h for phenotype analysis. (b) Time-course of *B. cinerea* infection of WT, V#12, and *MdWRKY75e* transgenic lines for 48 h for disease index analysis. WT: wild type; V#12: empty vector; *MdWRKY75d*#18, *MdWRKY75d*#25, and *MdWRKY75d*#36: *MdWRKY75d* transgenic lines.


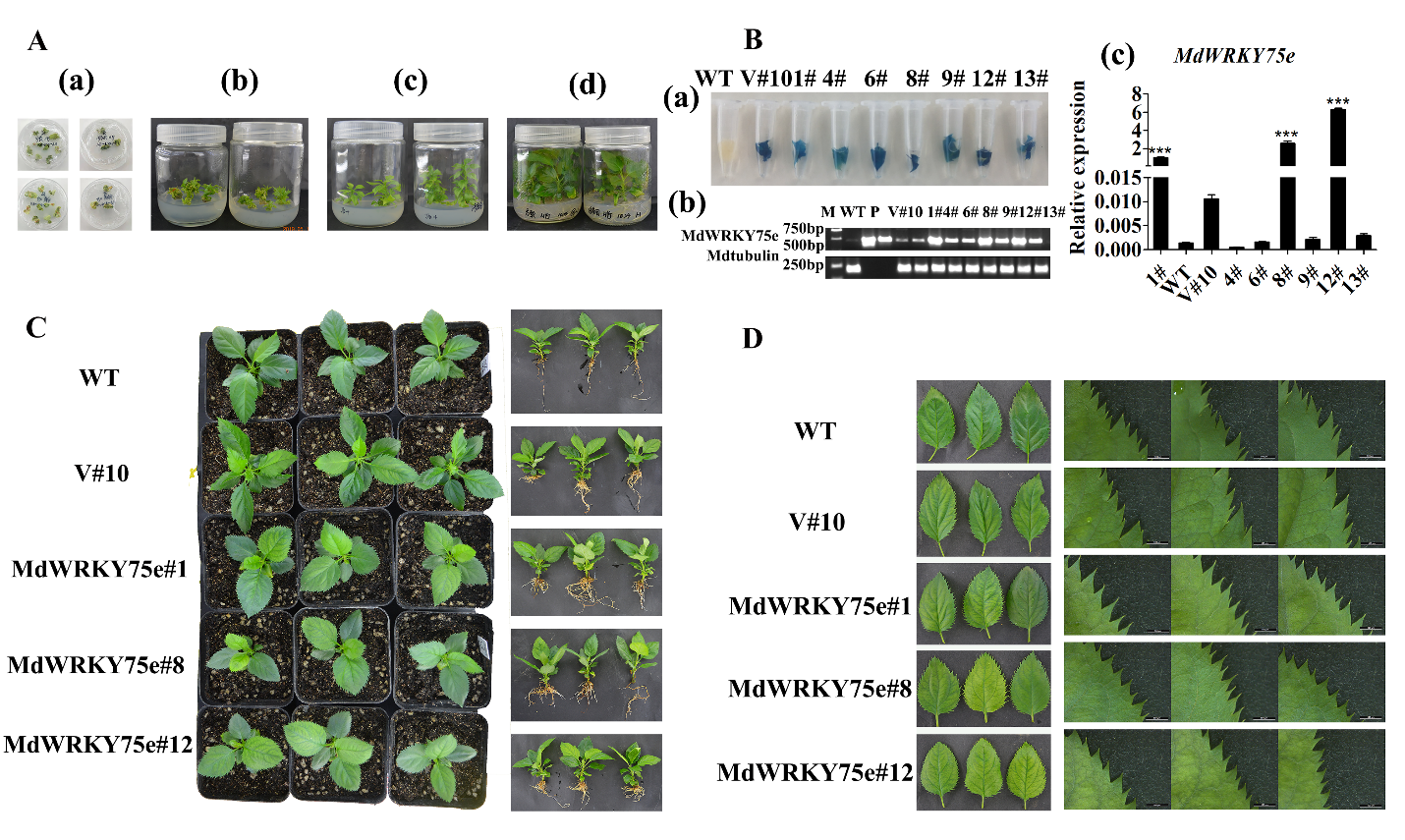


**Fig. S4** Generation and molecular identification of transgenic apple plants overexpressing *MdWRKY75e*. (A) *Agrobacterium*-mediated transformation of process of the apple leaf discs and plant regeneration(a-d). (B) GUS staining (a), semi-quantitative RT-PCR analysis (b), and expression of *MdWRKY75e* by qRT-PCR (c) in the wild type (WT), empty vector (V#10), and transgenic lines. M: DNA Marker; WT (wild type); P: plasmid; V#10: empty vector; the numbers and # indicated different transgenic lines. (C-D) Selected the plant (C), and leaf (D) phenotypes of transgenic lines. WT: wild type; V#10: empty vector; *MdWRKY75e*#1, *MdWRKY75e*#8, and *MdWRKY75e*#12: transgenic lines. The differences were analyzed by Tukey’s multiple range test. Different stars indicate significantly different (**P* < 0.05; ***P* < 0.01; ****P* < 0.001).


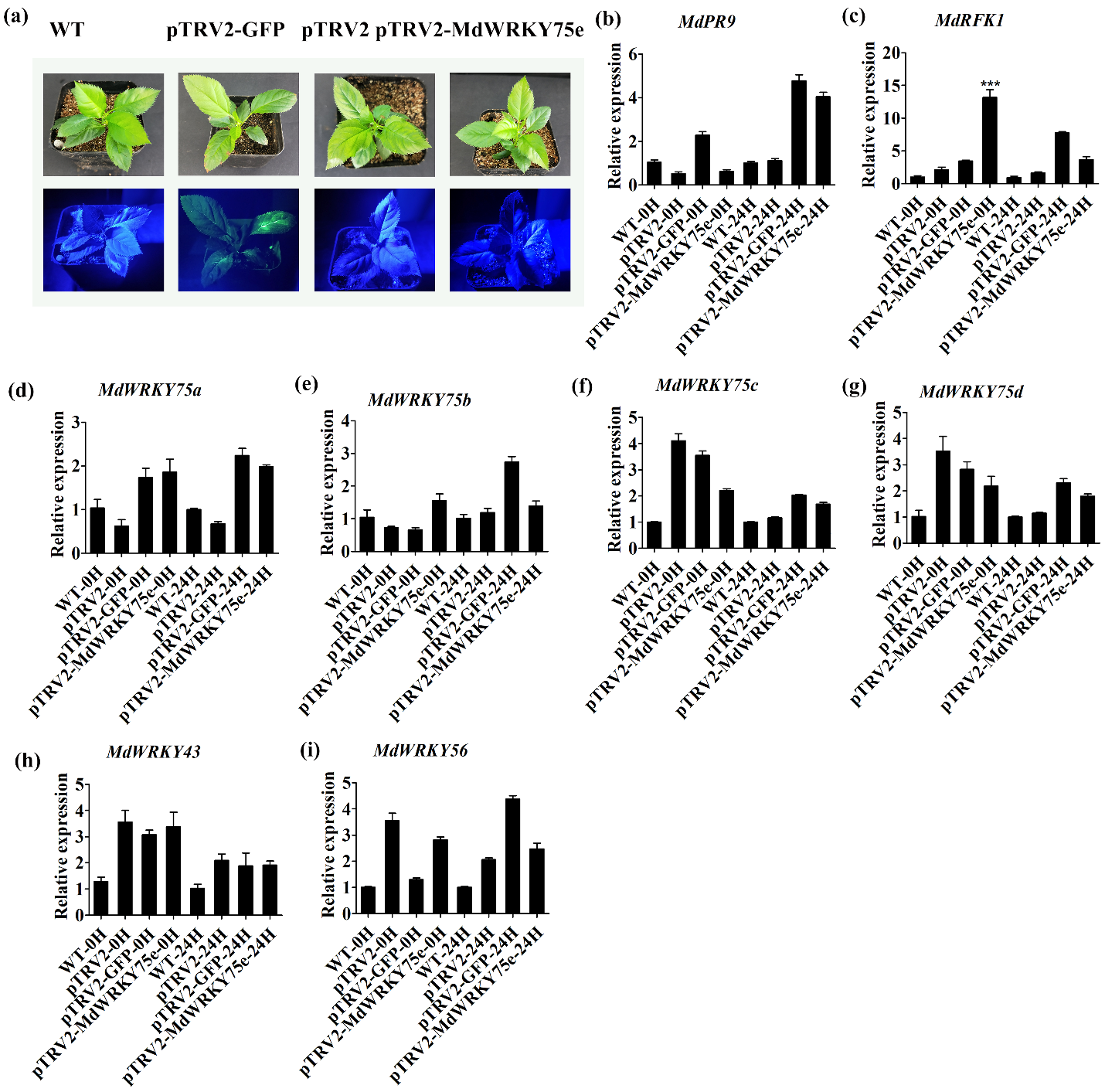


**Fig. S5** Verification of VIGS system and expression of MdWRKY75e target genes in apples. (a) Vector pTRV2-*GFP* to test and verify pTRV2 system in apples used for transient analysis. (b-c) Expression of MdWRKY75e target genes *MdPR9* (b), and *MdRFK1* (c). (d-i) Expression of MdWRKYs with the most evolutionary relationship to the *MdWRKY75e*. The differences were analyzed by Tukey’s multiple range test. Different stars indicate significantly different (**P* < 0.05; ***P* < 0.01; ****P* < 0.001).


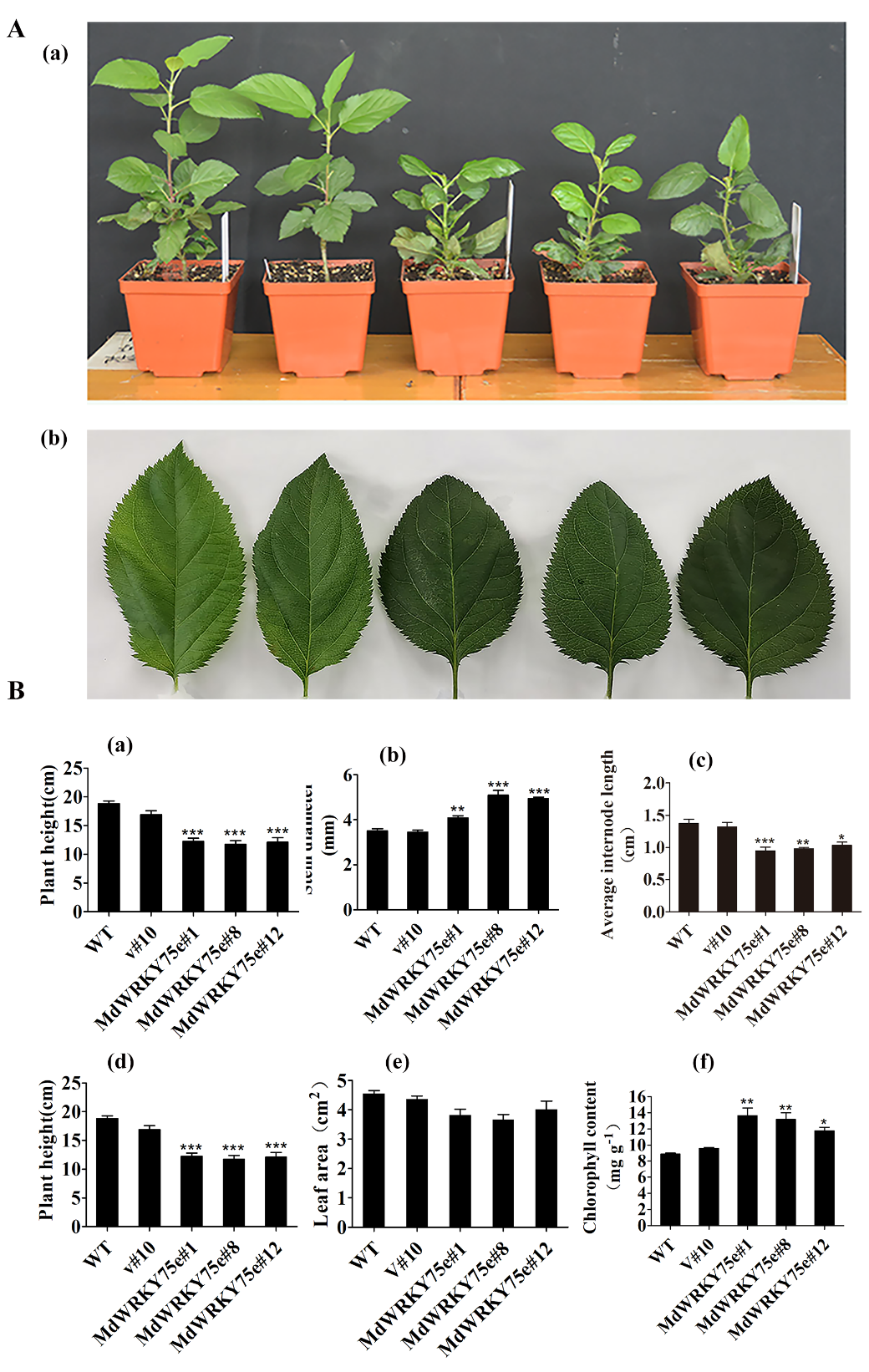


**Fig. S6** Overexpression of *MdWRKY75e* conferred enhanced dwarfing trait in transgenic apple. (A) The plant (a), and leaf (b) phenotypes of transgenic lines. (B) Results of Plant heigh (a), Stem diameter (b), Average internode length (c), Leaf index (d), Leaf area (e), and Chlorophyll content (f) in WT, V#10, and *MdWRKY75e* transgenic lines. WT: wild type; V#10: empty vector; *MdWRKY75e*#1, *MdWRKY75e*#8, and *MdWRKY75e*#12: *MdWRKY75e* transgenic lines. The data were analyzed by Tukey’s multiple range tests in the ANOVA program of SPSS (IBM SPSS 22). *, ** and *** indicate that values of the three transgenic lines were significantly different from those of WT and V#12 at *P*< 0.05, *P* < 0.01 and *P* < 0.001, respectively.


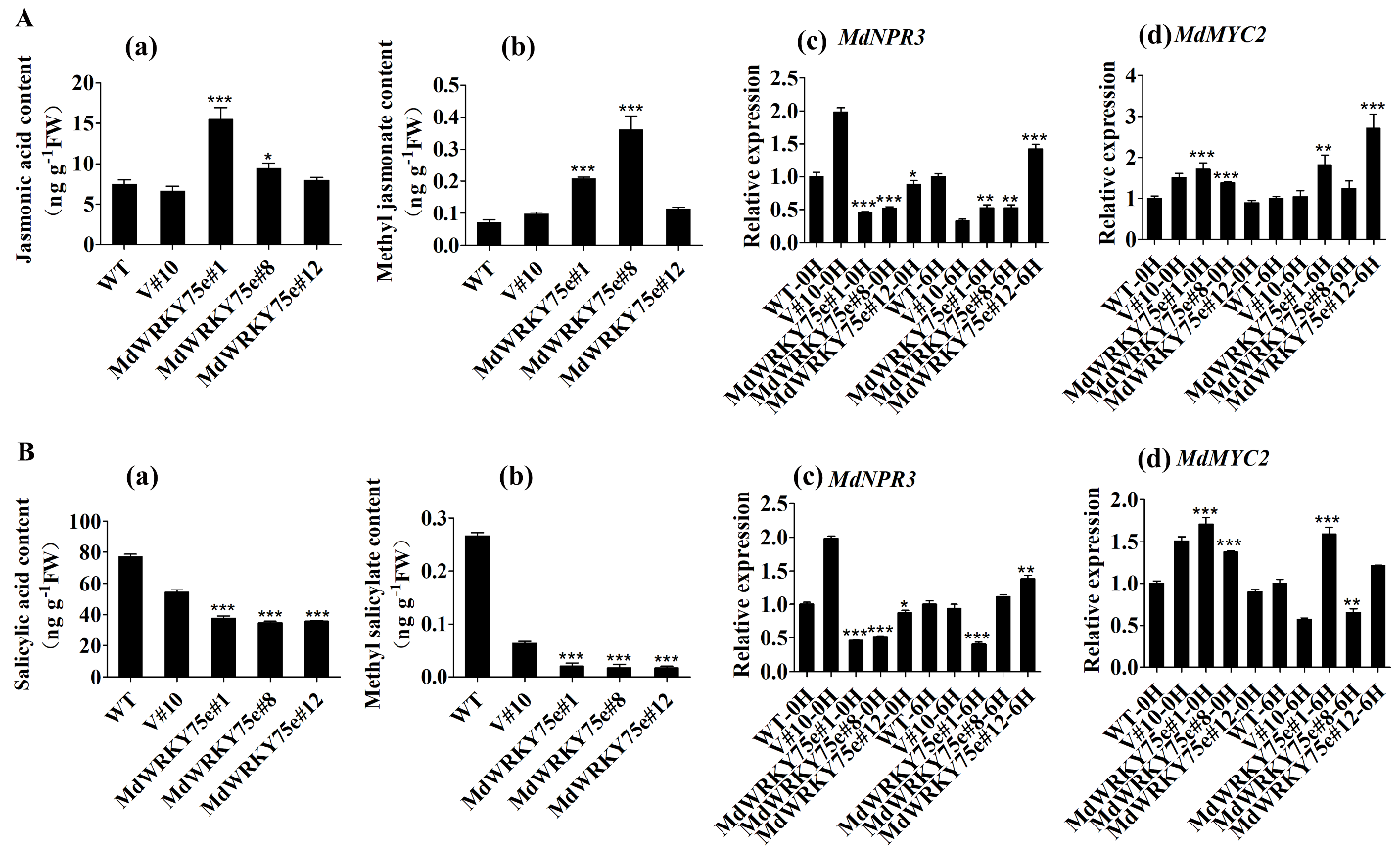


**Fig. S7** Levels of jasmonic acid (JA), salicylic acid (SA), and expression of related genes in WT, V#10, and *MdWRKY75e* transgenic lines. (A) Levels of jasmonic acid (JA) and expression of related genes in WT, V#10, and *MdWRKY75e* transgenic lines (a-d). (B) Levels of salicylic acid (SA) and expression of related genes in WT, V#10, and *MdWRKY75e* transgenic lines (a-d). WT: wild type; V#10: empty vector; *MdWRKY75e*#1, *MdWRKY75e*#8, and *MdWRKY75e*#12: *MdWRKY75e* transgenic lines. The differences were analyzed by Tukey’s multiple range test. Different stars indicate significantly different (**P* < 0.05; ***P* < 0.01; ****P* < 0.001).


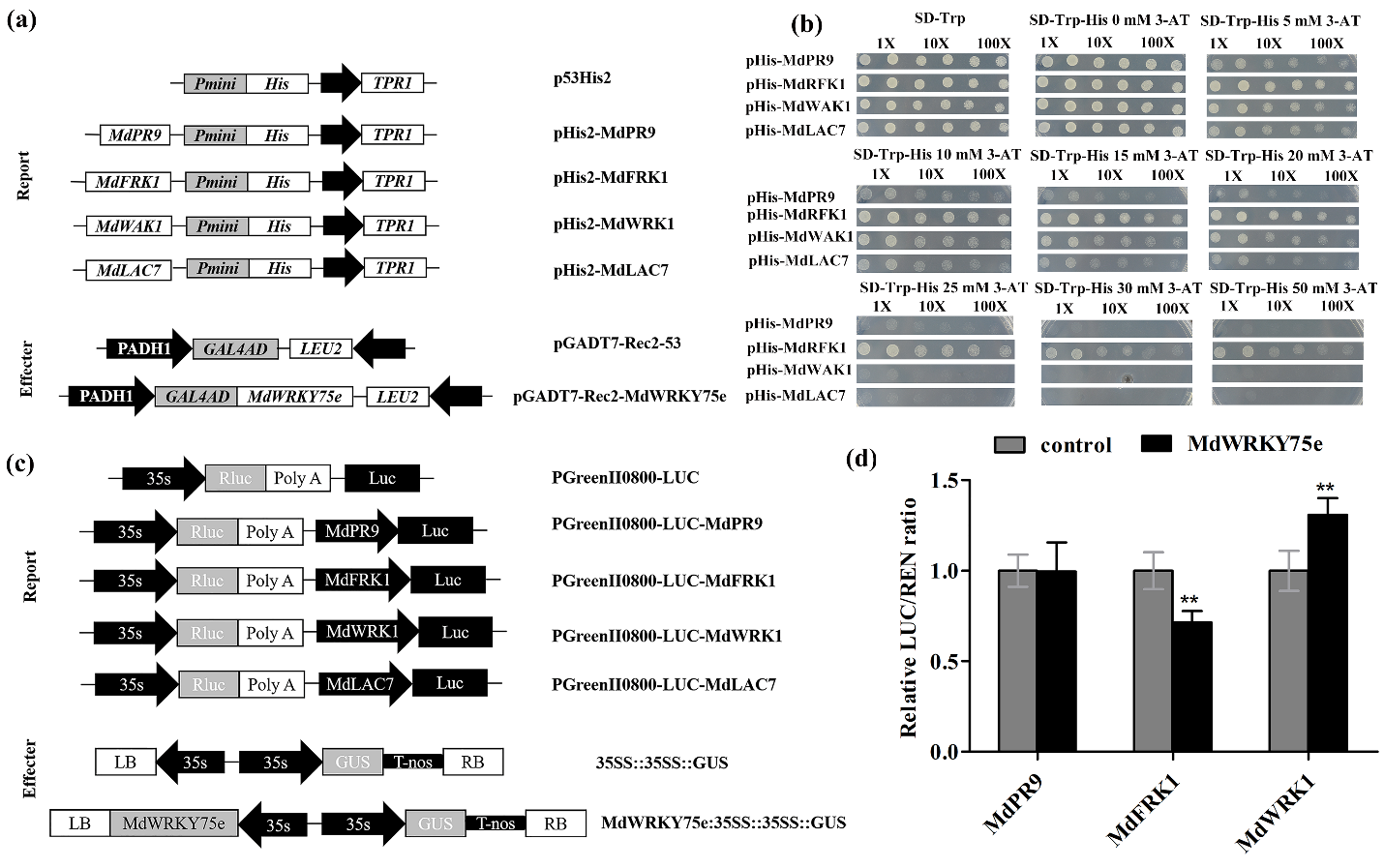


**Fig. S8** Schematic structures of vector and experimental results for yeast one-hybrid assay and instant expression analysis. (a) Schematic structures of the effector and reporter vector used for yeast one-hybrid assay. (b) Reporter vectors self-activating was filtered 3-AT inhibition concentration. (c) Illustration of the structures of the effector and reporter constructs employed for instant expression analysis. (d) Instant expression experiment of the activity of promoters co-converted with the effector and each of the reporters in tobacco leaves.

**Table S1** Primer sequences used for qRT-PCR in *Nicotiana tabacum* and *Malus domestica*.

| **Genes** | **Gene ID** | **Sequences (**5ʹ-3ʹ**)** | |
| --- | --- | --- | --- |
|  |  | **Forward** | **Reverse** |
| *MdWRKY75d* | MDP0000154734/XM_008380876.3 | CGATGTACTTGACGATGGCT | ACTTGCACCCTTGATACGTG |
| *MdWRKY75e* | MDP0000263768/XM_008376006.3 | TTCATCAGCATCCTCATCA | TGTTCACCTTATCACCTTCT |
| *NtPR1a/c* | XM_016581737.1 | AACCTTTGACCTGGGACGAC | GCACATCCAACACGAACCGA |
| *NtNPR1* | NM_001326267.1 | CTATCTTCGATGCGGCTTCTCC | CACCACAGCATCATAGCTCAC |
| *NtPR1b* | XM_016621637.1 | TTGGTTGTGCTAGGGTCAGG | TGCAAGATCACCGTAGGGAC |
| *NtHSR201* | NM_001325938.1 | CAGCAGTCCTTTGGCGTTGTC | GCTCAGTTTAGCCGCAGTTGTG |
| *NtHSR515* | NM_001325739.1 | TTGGGCAGAATAGATGGGTA | TTTGGTGAAAGTCTTGGCTC |
| *NtPR5* | XM_016609409.1 | TCGAGCGAGGTCAAAGCTGGT | CACAAGGTCCTTGTGTGCA |
| *NtACX1* | NM_001325883.1 | GAATGTCTGTTGCTTGTGCTCA | TACCGCAAAGCACCTCCAG |
| *NtSA-2* | NM_001325513.1 | TGATGGAGTTGATGGAATCT | GAGGAGTTGTGAACAGAATC |
| *NtJAR1* | XM_016636390.1 | GCCGTGTGACAGTTCCTTCT | TGTTCGGGAAAAGAGCTGGA |
| *Ntthil-2* | XM_016590793.1 | GTTTTTGGGAATGTTTGTAGGGCA | GCAGGTAGAAAAGGCACAACC |
| *NtPOD* | NM_001325975.1 | CTCCATTTCCATGACTGCTTTG | GTTGGGTGGTGAGGTCTTT |
| *NtSOD* | XM_016657042.1 | ACCACCAGAAGCATCATCAGACT | TAATGTGACCTCCGCCGTTG |
| *NtCAT* | NM_001325673.1 | TTCTGCCCTTCTATTGTGGTTCC | ATGAGCACACTTTGGAGCATTAGC |
| *NtPAL* | NM_001325544.1 | CTTGTCGACCACGCCTTAGA | AGGGTTCCCATTTTCCAGCG |
| *NtLAC7* | XM_016614910.1 | CGGGGTACCATTTGACAACAC | CCACTTTACGAGGTGGAGGGA |
| *MdPR1* | NM_001311210.1 | GCAGCAGTAGGCGTTGGTCCCT | CCAGTGCTCATGGCAAGGTTTT |
| *MdPR2* | XM_008368189.3 | CCTATGCCTTGTTCACTTC | TTGCTTGATTTCCACCTTC |
| *MdChit1* | NM_001293894.1 | TGGAGGATGGGAAAGTGC | GGGTGAGTTGGATGGGTC |
| *MdPR5* | XM_029097947.1 | AACTAGCATCCAAAGCTAGCC | CCACAGTCTGCAGTTTCACAAG |
| *MdNPR3* | NM_001293864.1 | GATTGTCTTCATCGTCCTCCAC | CAACATCTTCCTACGCTCTCG |
| *MdJA* | XM_029108725.1 | CGTTCCCTTCTCCACAATCC | GGGTTCTTGGTGAATGTTGC |
| *MdJAR4* | XM_029099236.1 | CAAGCACCTGAAAACGCGAG | CGATTGTTGGAAATCGGGGC |
| *MdPDF1* | XM_017333881.2 | TCTACCAAAGGCGGAGCAAA | ACTCCTGGGTATTGAGCGTG |
| *MdMYC2* | NM_001328944.1 | CTGGACCTACGCCATCTTCT | CTTGCGGTACTCTTGTTCGG |
| *MdWRKY75a* | XM_008391676.3 | CTCCTTCTTCCTTGTCATTG | CTAGTTCCAGATCCTCCAA |
| *MdWRKY75b* | XM_008343449.3 | ACGATGACGACAACAGAA | TCGGATAATGCTGCTGAT |
| *MdWRKY75c* | XM_008391676.3 | GAAGCAACTGTGAGATTAGG | TCCTCCATCTATATCCATCATC |
| *MdWRKY43* | XM_008355785.3 | AATGAGAAAAGCAGCGGCGA | TGTGATGTGTGCAGCGGTAG |
| *MdWRKY56* | XM_029104893.1 | AAGCGGTCAAGAACAGCTCA | TCACCCCCTTGGAACAACTC |
| *MdCAT* | XM_008375181.3 | CTCATCACAACAATCACCAT | TCTCAATGACGCACTTCT |
| *MdSOD* | XM_008372433.3 | GTTGACAAGCAGATTCCTC | CCAATAATACCGCAAGCC |
| *MdPAL* | XM_008389362.3 | TTGACGCACAAGTTGAAG | GAGGTGATGTTCGGAGAG |
| *MdPOD* | XM_008393504.3 | TTGACTGTACGAGGATACG | TGTTGGAGCAGCATAGTT |
| *MdLAC7* | MDP0000226556/XM_008389878.3 | TCATAATCCACCCGAAAGT | CCTTCTTCCTCAATGTCAAC |
| *MdPR9/POD* | XM_008354278.3 | ACCCTTGGGTCGTAGAGATG | TGAAGTGTCCAGTGTTGGGT |
| *MdWFK1* | MDP0000835304/XM_017332985.2 | CTCACTCTTCTTCTTGATC | GGATAGCGAAAGGTTAAG |
| *MdRFK1* | MDP0000554411/XM_029110295.1 | CCTATCTCCGCTTTCACT | GCCATACCTGCCTCATAT |
| *Alt a 1* | XM_028652247.1 | TCCGAATTCTACGGACGCAA | TGAGCAGAGCAGGTGAAGTC |
| *GAPdh* | XM_028650964.1 | ATGGGTGTCAACCACGAGAC | CCTTCTGGGTGGCAGTGTAG |
| *NtTubulin* | NM_001325310.1 | AGATGTTCCGTCGTGTCAGTG | TGCTTCCTCTTCATCCTCATATCC |
| *MdTubulin* | XM_008378370.3 | AGGATGCTACAGCCGATGAG | GCCGAAGAACTGACGAGAATC |

**Table S2** Primer sequences used for cloning and vector construction.

| **Genes** | **Vector** | **Sequences (**5ʹ-3ʹ**)** | |
| --- | --- | --- | --- |
|  |  | **Forward** | **Reverse** |
| *MdWRKY75d* |  | GCTACATCAACCCTAGATACCCC | CTTTTACAAAAGCAAGCAAGCA |
| *MdWRKY75e* |  | TCCTCTCAGATGGATCACAACAA | GAAGCTTTAATTATTAGGCACAGT |
| *MdWRKY75d* | pCAMBIA1301 | GAGCTCATGGAGAAGTATCAAATGTT (*SacI* site is underlined) | GGATCCTCAGATGTGGGTGTAAATTTGCATC (*BamHI* site is underlined) |
| *MdWRKY75e* | pCAMBIA1301 | GAGCTCATGGATCACAACAACCAAAT (*SacI* site is underlined) | GGATCCTTAATTATTAGGCACAGTAGATGCT (*BamHI* site is underlined) |
| *MdWRKY75e* | pCAMBIA2300 | GTCGACATGGATCACAACAACCAAAT (*SalI* site is underlined) | TCTAGATTAATTATTAGGCACAGTAGATGCT (*XbaI* site is underlined) |
| *MdWRKY75d* | pGBKT7 | CCATGGGCATGGAGAAGTATCAAATGTTCTTTC (*NcoI* site is underlined) | GGATCCGATGTGGGTGTAAATTTGCATC (*BamHI* site is underlined) |
| *MdWRKY75e* | pGBKT7 | CCATGGGCATGGATCACAACAACCAAAT (*NcoI* site is underlined) | GGATCCATTATTAGGCACAGTAGATGCT (*BamHI* site is underlined) |
| *MdWRKY75d* | pCAMBIA1302 | CCATGGGCATGGAGAAGTATCAAATGTTCTTTC (*NcoI* site is underlined) | ACTAGTGATGTGGGTGTAAATTTGCATC (*SpeI* site is underlined) |
| *MdWRKY75e* | pCAMBIA1302 | CCATGGGCATGGATCACAACAACCAAAT (*NcoI* site is underlined) | ACTAGTATTATTAGGCACAGTAGATGCT (*SpeI* site is underlined) |
| *MdWRKY75e* |  | CTTCCTTGGTTCATCAGCATCCTCA | AAGTATGTCAACCTGGCTCCTTGTT |
| *MdWRKY75e* | pTRV2 | GAATTCCTTCCTTGGTTCATCAGCATCCTCA (*EcoRI* site is underlined) | CTCGAGAAGTATGTCAACCTGGCTCCTTGTT (*XhoI* site is underlined) |
| *MdWRKY75e* | pGBKT7-Rec | GAATTCATGGATCACAACAACCAAAT  (*EcoRI* site is underlined) | CTCGAGTTAATTATTAGGCACAGTAGATGCT (*XhoI* site is underlined) |
| *MdLAC7* |  | GAATGCCAAGGCAAAGTCGAT | TCAACAATTGCACCAGAGGC |
| *MdPR9* |  | TTGACCGAACAAAGTGGATTAAAGA | GCATCTATGGCTTGTGCTGG |
| *MdWFK1* |  | AGTGCCAAAGGAGTTGGGAA | CATCAGCAGAGTGGGAGACT |
| *MdRFK1* |  | CAACAGCTACTGATGCAACGC | CGGGTGTGAAACTGTGATTGC |
| *MdLAC7* | pHis2 | GAATTCGAATGCCAAGGCAAAGTCGAT (*EcoRI* site is underlined) | GAGCTCTCAACAATTGCACCAGAGGC (*SacI* site is underlined) |
| *MdPR9* | pHis2 | GAATTCTTGACCGAACAAAGTGGATTAAAGA (*EcoRI* site is underlined) | GAGCTCGCATCTATGGCTTGTGCTGG (*SacI* site is underlined) |
| *MdWFK1* | pHis2 | GAATTCAGTGCCAAAGGAGTTGGGAA (*EcoRI* site is underlined) | GAGCTCCATCAGCAGAGTGGGAGACT (*SacI* site is underlined) |
| *MdRFK1* | pHis2 | GAATTCCAACAGCTACTGATGCAACGC (*EcoRI* site is underlined) | GAGCTCCGGGTGTGAAACTGTGATTGC (*SacI* site is underlined) |
| *MdLAC7* | pGreen II 0800-LUC | AAGCTTGAATGCCAAGGCAAAGTCGAT (*HindIII* site is underlined) | GGATCCTCAACAATTGCACCAGAGGC (*BamHI* site is underlined) |
| *MdPR9* | pGreen II 0800-LUC | AAGCTTTTGACCGAACAAAGTGGATTAAAGA (*HindIII* site is underlined) | GGATCCGCATCTATGGCTTGTGCTGG (*BamHI* site is underlined) |
| *MdWFK1* | pGreen II 0800-LUC | AAGCTTAGTGCCAAAGGAGTTGGGAA (*HindIII* site is underlined) | GGATCCCATCAGCAGAGTGGGAGACT (*BamHI* site is underlined) |
| *MdRFK1* | pGreen II 0800-LUC | AAGCTTCAACAGCTACTGATGCAACGC (*HindIII* site is underlined) | GGATCCCGGGTGTGAAACTGTGATTGC  (*BamHI* site is underlined) |
| *MdWRKY75e* | pMAL-c5X | atgtccatgggcggccgcATGGATCACAACAACCAAA | attacctgcagggaattctTTAATTATTAGGCACAGTA |
| *MdLAC7-*Probe |  | CCATGCGTA**TTGACC**ACGACAGCTT | AAGCTGTCGTGGTCAATACGCATGG |
| *MdLAC7-*mProbe |  | CCATGCGTA**TCGAAC**ACGACAGCTT | AAGCTGTCGTGTTCGATACGCATGG |

**Table S3** Analysis of disease resistance responsive genes promoter W-box element.

| Gene | GeneBank ID | related element | Quantity |
| --- | --- | --- | --- |
| *MdPR9* | XM_008354278.3 | W-BOX(TTGACC) | 1 |
| *MdWFK1* | XM_017332985.2 | W-BOX(TTGACC) | 2 |
| *MdRFK1* | XM_029110295.1 | W-BOX(TTGACC) | 1 |
| *MdLAC7* | XM_008389878.3 | W-BOX(TTGACC) | 1 |
